# Elemental superdoping of graphene and carbon nanotubes

**DOI:** 10.1038/ncomms10921

**Published:** 2016-03-04

**Authors:** Yuan Liu, Yuting Shen, Litao Sun, Jincheng Li, Chang Liu, Wencai Ren, Feng Li, Libo Gao, Jie Chen, Fuchi Liu, Yuanyuan Sun, Nujiang Tang, Hui-Ming Cheng, Youwei Du

**Affiliations:** 1 Nanjing National Laboratory of Microstructures, Nanjing University, Nanjing 210093, China; 2 Collaborative Innovation Center of Advanced Microstructures, Nanjing University, Nanjing 210093, China; 3 FEI Nano-Pico Center, Key Laboratory of MEMS of Ministry of Education, Collaborative Innovation Center for Micro/Nano Fabrication, Device and System, Southeast University, Nanjing 210096, China; 4 Advanced Carbon Division, Shenyang National Laboratory for Materials Science, Institute of Metal Research, Chinese Academy of Sciences, 72 Wenhua Road, Shenyang, Liaoning Province 110016, China

## Abstract

Doping of low-dimensional graphitic materials, including graphene, graphene quantum dots and single-wall carbon nanotubes with nitrogen, sulfur or boron can significantly change their properties. We report that simple fluorination followed by annealing in a dopant source can superdope low-dimensional graphitic materials with a high level of N, S or B. The superdoping results in the following doping levels: (i) for graphene, 29.82, 17.55 and 10.79 at% for N-, S- and B-doping, respectively; (ii) for graphene quantum dots, 36.38 at% for N-doping; and (iii) for single-wall carbon nanotubes, 7.79 and 10.66 at% for N- and S-doping, respectively. As an example, the N-superdoping of graphene can greatly increase the capacitive energy storage, increase the efficiency of the oxygen reduction reaction and induce ferromagnetism. Furthermore, by changing the degree of fluorination, the doping level can be tuned over a wide range, which is important for optimizing the performance of doped low-dimensional graphitic materials.

## Abstract (web summary)

Doping of low-dimensional graphitic materials with heteroatoms can enhance their catalytic, electrochemical and magnetic properties. Here, the authors report a tunable method to ‘superdope' these materials with high levels of nitrogen, sulfur, or boron, via a simple fluorination and annealing procedure.

## Graphical abstract

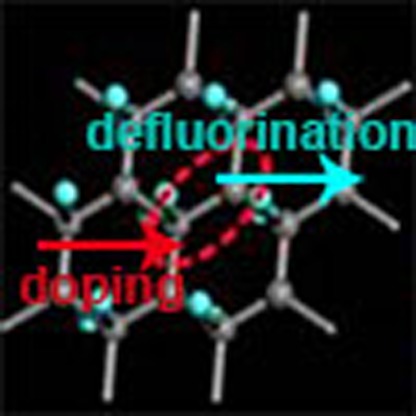

Low-dimensional graphitic materials (LDGMs) such as two-dimensional graphene sheets, one-dimensional single-wall carbon nanotubes (SWCNTs) and zero-dimensional graphene quantum dots (GQDs) show many properties that are quite different from those of bulk graphite and these allow them to be used in a wide range of applications^1^,^2^,^3^,^4^,^5^,^6^. It has been demonstrated that doping the graphitic lattice of LDGMs with heteroatoms such as N, S or B can change their physical and chemical properties, and significantly increase their number of applications^7^,^8^,^9^,^10^,^11^,^12^,^13^,^14^,^15^,^16^,^17^,^18^,^19^,^20^,^21^,^22^,^23^,^24^,^25^,^26^,^27^,^28^,^29^,^30^,^31^,^32^,^33^,^34^,^35^.

Numerous studies have shown that a high doping level is often needed to obtain LDGMs with the desired properties^23^,^24^,^25^,^26^,^27^,^28^,^29^,^30^,^31^,^32^. To date, various methods have been developed for the doping of LDGMs with N, S or B. In general, there are two different doping processes: (i) doping during their synthesis and (ii) doping post synthesis. Among these, thermal annealing is a universal postsynthesis approach, whereby doping can be realized by annealing the LDGMs in the appropriate dopant sources. Compared with other methods, this has some distinct advantages such as its high doping efficiency (a higher doping level and a wider level of adjustment than by using other methods), its wide applicability to LDGMs of any dimensions (graphene, GQDs or SWCNTs) and forms (powders, thin films or single nanodevices), and its ability to recover the *sp*^2^ network by thermal annealing^29^ and so on.

As these heteroatoms are mainly doped by entering vacancies in the graphitic lattice, their doping level is often limited by the low concentration of vacancies in LDGMs^32^. For high-level doping, creating vacancies in the graphitic lattice is key and this has been achieved by oxidation and subsequent de-oxidation^8^, NH_3_ etching^31^ or ion irradiation^36^. Even so, the maximum doping levels (defined as N/C, S/C and B/C × 100 at% for N-, S- and B-doping, respectively) reported by the thermal annealing of LDGMs are quite low^29^,^30^,^31^. For graphene, the maximum values for N, S and B doping are 14.68, 1.7 and 3.64 at%, respectively. For GQDs, the maximum N-doping level is 5.0 at%. For SWCNTs, the N-doping level of 5.0 at% has been realized via *in situ* doping^16^. In contrast, almost no N- or S-doping is possible in SWCNTs by postsynthesis doping. Therefore, achieving a high doping level in LDGMs remains a critical challenge^29^,^30^,^31^,^32^.

Graphene doped with a high-level of N is highly desirable for high-performance supercapacitors, fuel cells, energy-storage devices, ferromagnetic materials^6^,^29^,^30^,^31^ and so on. For example, the specific capacitance of pristine graphene can be increased from 69 to 280 F g^−1^ by doping with 2.51 at% N^37^ and to 326 F g^−1^ by doping with 10.13 at% N^38^. Pristine graphene lacks catalytic activity in the oxygen reduction reaction (ORR)^39^ and it is believed that N-doping can transform pristine graphene to an effective metal-free electrocatalyst for the ORR, and that a higher N level results in a higher ORR activity^40^. N-doping of graphene to 8.8 at% induces ferromagnetism with a high magnetization of 1.66 emu g^−1^ and a Curie temperature (*T*_C_) of 100.2 K (ref. 26).

In this study, we show that by fluorination followed by thermal annealing, it is possible to dope all three types of LDGMs with very high levels of N, S or B. The maximum doping levels obtained for graphene are 29.82 at% for N-doping (NG), 17.55 at% for S-doping (SG) and 10.79 at% for B-doping (BG). These are respectively 2, 10 and 3 times higher than the maximum levels previously reported. For GQDs the maximum N doping level is 36.38 at% (N-GQDs), 7 times higher than the reported maximum level, and for SWCNTs the maximum N and S doping levels are respectively 7.79 (N-SWCNTs) and 10.66 at% (S-SWCNTs), which are significantly higher than the near-zero levels currently reported. These doping amounts can be easily and precisely tuned over a very wide range by changing the degree of fluorination. We therefore call this highly efficient fluorination-assisted doping method ‘superdoping'. In addition, we report some excellent properties of superdoped NG, such as its high capacitance (390 F g^−1^), superior ORR properties (onset potential of −0.05 eV and a current density of −4.99 mA cm^−2^) and near room-temperature (RT) ferromagnetism (a high magnetization of 2.3 emu g^−1^ and a high *T*_C_ of 250 K).

## Results

### Superdoping of LDGMs with N

We doped LDGMs with N by fluorination followed by annealing in ammonia. As N atoms mainly enter the graphite lattice through vacancies, creating vacancies in the lattice is necessary to achieve high levels of N-doping. It is known that carbon monofluoride is unstable at high temperatures^41^, and that thermal defluorination of fluorinated LDGMs (F-LDGMs) by annealing consumes C atoms. Thus, it is reasonable to speculate that fluorination followed by thermal defluorination will (i) generate a high concentration of vacancies in the graphitic lattice, which will stimulate high-level N-doping and (ii) allow one to tune the vacancy concentration by changing the degree of fluorination, which may result in an ability to tune the N-doping level. In other words, one can dope LDGMs with a very high level of N by fluorination followed by thermal defluorination, and also can tune the N-doping level over a very wide range by changing the degree of fluorination. We explored this phenomenon using graphene, GQDs and SWCNTs (Supplementary Figs 1–3).

**Figure 1:**
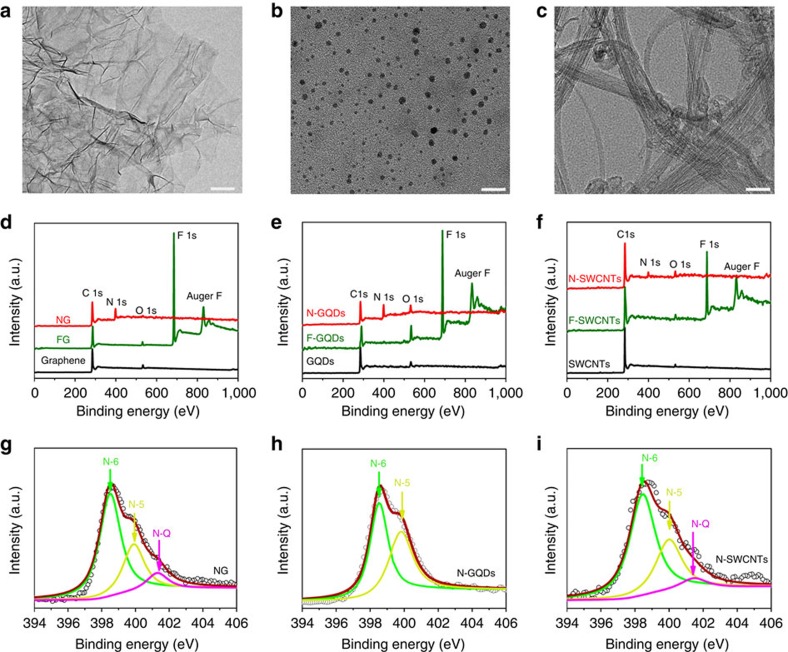
Superdoping of LDGMs with N. TEM images of (**a**) graphene, (**b**) GQDs and (**c**) SWCNTs. Scale bars, 500 nm (**a**); 20 nm (**b**); 50 nm (**c**). XPS spectra over a wide range of binding energies of (**d**) graphene, FG and NG; (**e**) GQDs, F-GQDs and N-GQDs; and (**f**) SWCNTs, F-SWCNTs and N-SWCNTs. The fine-scanned N 1 s XPS spectra of (**g**) NG, (**h**) N-GQDs and (**i**) N-SWCNTs. The dots are measured data and the solid lines are fitted curves.

To study the structural transformation of LDGMs during fluorination and annealing in ammonia vapour, we carefully characterized pristine, fluorinated and N-doped LDGMs (N-LDGMs) by transmission electron microscopy (TEM), X-ray photoelectron spectroscopy (XPS) and Raman spectroscopy. It was found that, for the original materials, (i) the graphene consists of a few sheets of micrometre lateral size (Fig. 1a) and has a low oxygen content of 4.31 at% (Fig. 1d); (ii) GQDs are mainly distributed in the size range 1–7 nm and have a high crystallinity (Fig. 1b and Supplementary Fig. 1a,b) and an oxygen content of 5.24 at% (Fig. 1e); and (iii) the SWCNTs have diameters of 1–2 nm (Fig. 1c and Supplementary Fig. 2). For fluorination, the LDGMs were heated in XeF_2_ (ref. 42). Based on the XPS results of the F-LDGMs (Fig. 1d–f), we can calculate the fluorination degrees (defined as F/C × 100 at%). Both fluorinated graphene (FG) and fluorinated GQDs (F-GQDs) have high fluorination degrees of 103.52 and 102.04 at%, respectively. In contrast, fluorinated SWCNTs (F-SWCNTs) have a relatively low fluorination degree of 34.24 at%.

We found that the optimum annealing temperature to obtain high-level doping of FG in ammonia is 500 °C (Supplementary Table 1) and this is therefore used as the annealing temperature for F-GQDs and F-SWCNTs. After annealing in ammonia, the disappearance of the F peak is accompanied by the appearance of a clear N peak at ∼400 eV in all three N-LDGMs, indicating complete defluorination and effective N-doping (Fig. 1d–f). This can also be confirmed by fine scans of the C 1 s XPS spectra (Supplementary Fig. 4). Based on the XPS results (Fig. 1d–f), we calculated the N-doping levels to be 29.82, 36.38 and 7.79 at% for NG, N-GQDs and N-SWCNTs, respectively. The N level of N-GQDs is higher than that of NG, which may be attributed to the larger number of edge sites for N-doping in the GDQs. The N contents of NG and N-GQDs are two and seven times the corresponding maximum values reported^29^,^30^,^31^. In contrast, the N level of N-SWCNTs is relatively low, which may result from the relatively low fluorination degree obtained. However, one should note that the N-doping level obtained by the annealing of SWCNTs in ammonia is almost undetectable in the present studies. It is reasonable to assume that the N-doping level in SWCNTs can be further increased if one can increase the fluorination degree.

Fine scans of the N 1s XPS spectra of the three N-LDGMs were performed and deconvoluted into three sub-peaks located at ∼398.3, 400.0 and 401.4 eV (Fig. 1g–i), which may be respectively attributed to pyridinic N (N-6), pyrrolic N (N-5) and graphitic-like N (N-Q) types^29^,^30^,^31^,^32^. It is found that N-6 and N-5 dominate the N-doping in all three N-LDGMs (Supplementary Table 2), similar to the reported results from N-LDGMs produced by annealing in ammonia. Based on the fact that both N-6 and N-5 are mainly doped in vacancies in the graphitic lattice^29^,^30^,^31^, their high levels indicate a high concentration of vacancies in N-LDGMs. We directly annealed the pristine LDGMs in ammonia and found that the N levels are low, with values of 4.91, 10.18 and 0.67 at% for NG', N-GQDs' and N-SWCNTs' (Supplementary Fig. 5), respectively. This implies that the vacancy concentrations of the three original LDGMs are low, and that the fluorination/defluorination process generates a high concentration of vacancies, which facilitate N-doping.

### Demonstration of uniformly distributed N atoms in NG sheets

**Figure 2:**
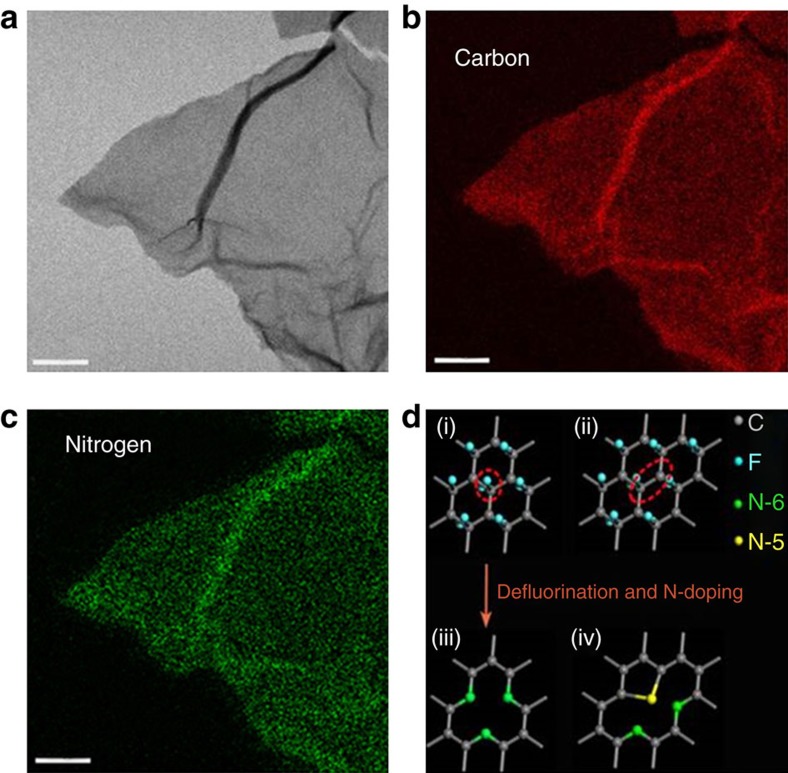
Demonstration of uniformly distributed N atoms in NG sheets. (**a**) Energy-filtered TEM (EFTEM) image (zero loss) of the NG sheet. (**b**,**c**) EFTEM elemental mapping images obtained by using electron energy loss (EEL) edges of C (284 eV, depicted in red in **b**), and N (401 eV, depicted in green in **c**). Scale bars, 100 nm. (**d**) Schematic of defluorination and N-doping of a fluorinated graphite lattice. The red ring and elliptical ring in the fluorinated graphite lattice indicate that a mono- (i) and di-vacancy (ii) are generated after thermal defluorination. Three N-6 atoms (iii), and two N-6 and one N-5 atoms (iv) are generated by substituting three C atoms at the mono- and di-vacancy, respectively.

A uniform distribution of N atoms in the graphite lattice is important to achieve the properties of N-doped LDGMs needed for new applications. We carefully investigated the distribution of N atoms in NG by energy-filtered TEM in an image aberration-corrected TEM. As shown in Fig. 2a–c, it is found that the N atoms are uniformly distributed in the NG sheet, indicating that uniformly distributed vacancies were generated after the thermal defluorination of FG. It is known that, as carbon monofluoride is unstable at a high temperature, thermal defluorination of FG can consume C and F atoms instead of only F atoms, and thus introduce vacancies in the graphite lattice^41^. It is considered that a mono-vacancy can contribute three N-6 atoms (left of Fig. 2d) and a di-vacancy can introduce two N-6 atoms and one N-5 atom (right of Fig. 2d)^31^. Considering the fact that the levels of N-6 and N-5 are very high and the ratio of N-5/N-6 is 0.5 for NG, one can suggest that thermal defluorination of a fluorinated graphite lattice in ammonia generates a high-concentration di-vacancies, which significantly stimulate N-doping.

An *in situ* investigation of the simultaneous steps of vacancy generation by thermal defluorination and N-doping is difficult. We divided the process into two separate steps: the first is defluorination of FG in Ar, to obtain defluorinated graphene, and the second is N-doping of defluorinated graphene by annealing in ammonia, to obtain NG''. It is surprising that the N levels of NG'' are as high as 21.03, 13.23 and 6.61 at% for total N, N-6 and N-5 (Supplementary Fig. 6 and Supplementary Table 3), respectively. This indicates that thermal defluorination of FG in Ar generates a high concentration of vacancies and thus facilitates N-doping. However, the total N level of NG'' is only ∼66.5% of the value of NG by fluorination followed by annealing, indicating that *in situ* N-doping during thermal defluorination is important in achieving the high N-doping level. The lower amount of N in NG'' may be attributed to the fact that *in situ* N-doping restrains the reconfiguration of defluorination-generated di-vacancies.

### Superdoping of graphene and SWCNTs with S or B

**Figure 3:**
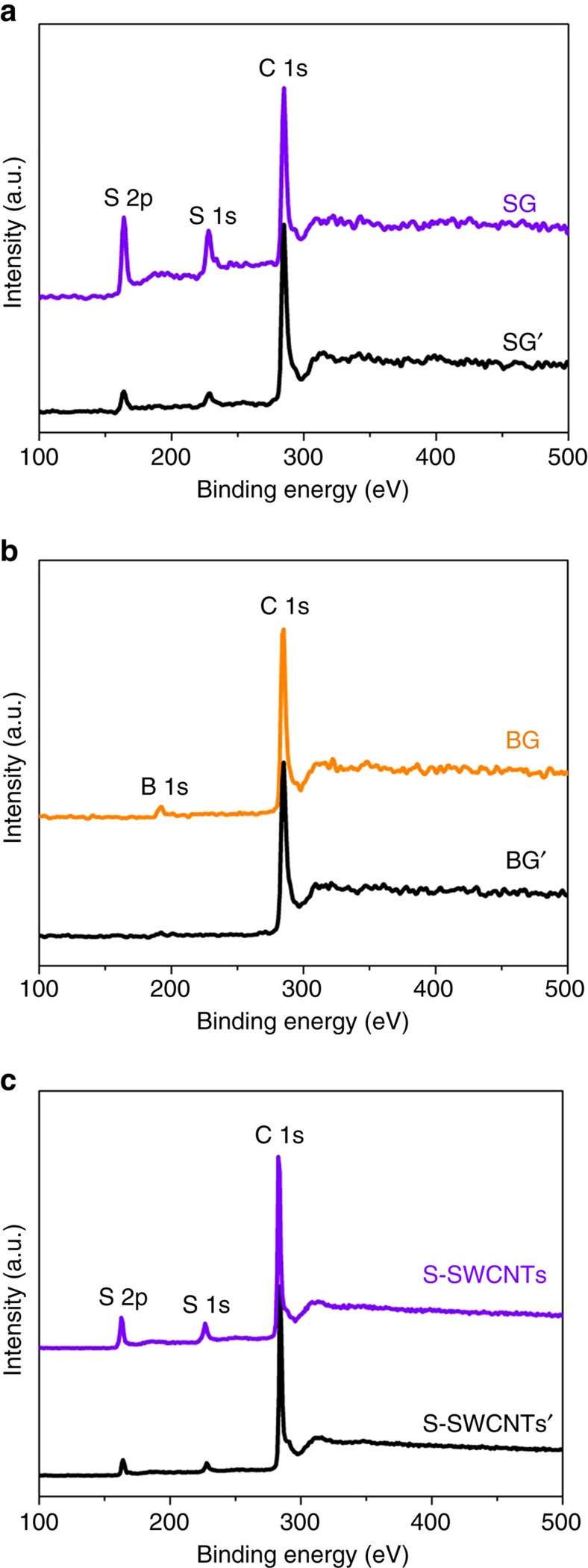
Superdoping of graphene and SWCNTs with S or B. XPS spectra over a wide range of binding energies of (**a**) SG obtained by fluorination followed by annealing in sulfur vapour and SG' by annealing in sulfur vapour; (**b**) BG obtained by fluorination followed by annealing in BCl_3_ vapour and BG′ obtained by annealing in BCl_3_ vapour; and (**c**) S-SWCNTs obtained by fluorination followed by annealing in sulfur and S-SWCNTs by annealing in sulfur vapour.

There is no doubt that both S- and B-doping are less effective than N-doping of graphene^9^,^43^. To investigate the scalability of our superdoping method, we doped graphene with S (SG) and with B (BG) by this method (see Methods for details of synthesis methods). For comparison we directly doped graphene with S (SG') by annealing it in sulfur vapour and with B (BG′) by annealing it in BCl_3_ vapour (see Methods for details of synthesis methods). It was found that, compared with direct annealing the combination of fluorination and annealing resulted in significant increases in the doping levels. These are from 4.66 to 17.55 at% for S and from 3.2 to 10.79 at% for B. It was also found that SG contains sulfur atoms that are doped into the graphite lattice, forming N-6- and N-5-like structures with neighbouring carbon atoms (Supplementary Fig. 7). We also doped SWCNTs with S by fluorination followed by annealing in sulfur vapour (S-SWCNTs) and by directly annealing in sulfur vapour (S-SWCNTs'), and again observed a drastic increase in the S level (from 3.49 to 10.66 at%, calculations based on Fig. 3c). It is clear that both the S- and B-doping levels of LDGMs by our method are much higher than the maximum values previously reported^17^,^19^,^20^,^21^,^22^,^23^,^29^,^30^,^31^,^32^. All these results demonstrate the wide suitability of this fluorination-followed-by-annealing method for the superdoping of LDGMs. In view of the fact that both S- and B-doping occur at vacant lattice sites in the graphite lattice of LDGMs, the high doping levels of both S and B further confirm that the thermal defluorination of F-LDGMs can generate a high-concentration of vacancies and facilitate doping.

### Adjusting doping levels by varying the fluorination degree

**Figure 4:**
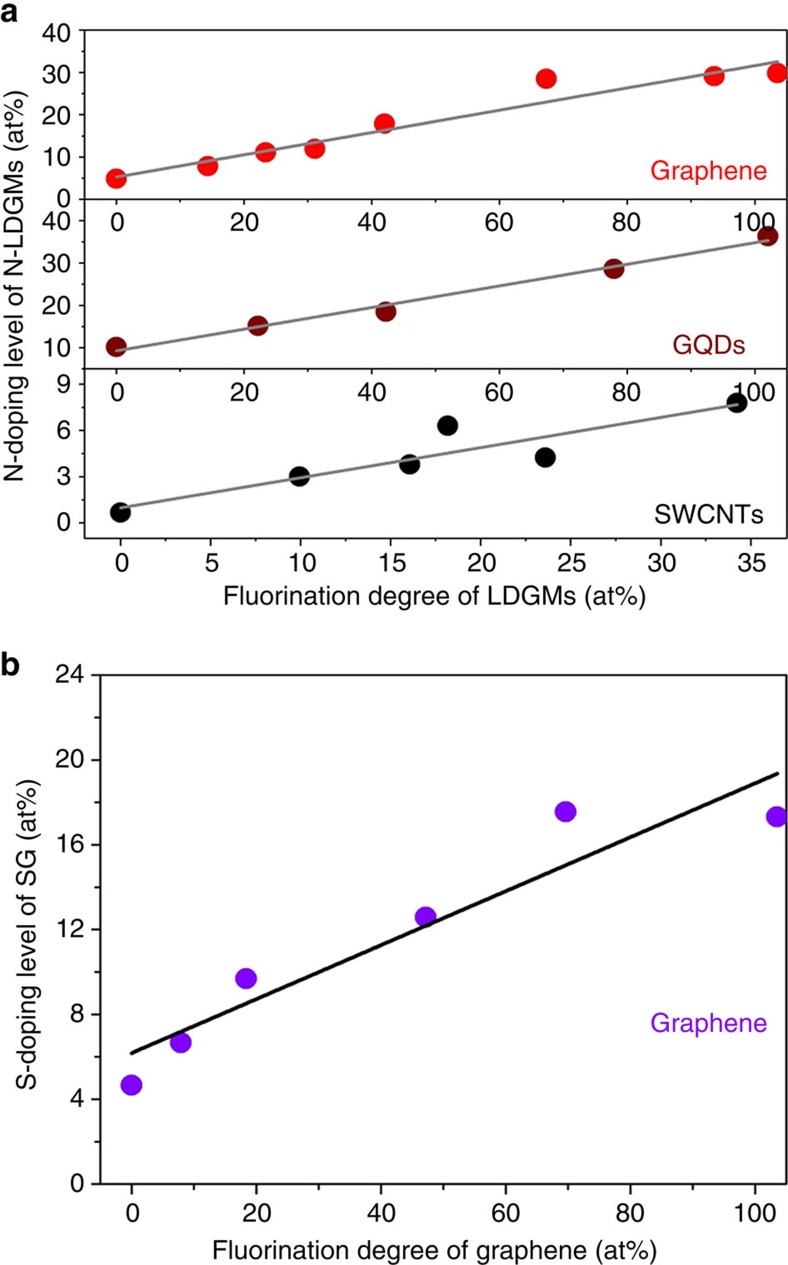
Adjustability of the N- and S-doping levels by varying the fluorination degrees of LDGMs. (**a**) Dependence of the N-doping level on the fluorination degree of LDGMs. (**b**) Dependence of the S-doping level on the fluorination degree of graphene.

Both the N- and S-doping levels of LDGMs are generally so low that it is virtually impossible to control the doping level. To synthesize FG, F-GQDs and F-SWCNTs with different fluorination degrees, we changed the mass ratio of LDGMs to XeF_2_. From the XPS data on the F-LDGMs and N-LDGMs (Supplementary Fig. 8 and Supplementary Tables 4–6), we obtained the dependence of the N-doping level on the fluorination degree of the LDGMs. The results show that by varying the fluorination degree the corresponding N level can be adjusted over a wide range. For graphene this was from 4.91 to 29.82, whereas for GQDs it was from 10.18 to 36.38, and for SWCNTs from 0.67 to 7.79 at% (Fig. 4a). Similar experiments to obtain the dependence of the S-doping level on the fluorination degree of graphene (see Methods for details about synthesis methods) showed that it could be adjusted from 4.66 to 17.55 at% (Fig. 4b). For both N-LDGMs and SG, the doping level increases almost linearly with the increase of the fluorination degree. These results indicate that the doping levels of N, S or B can be precisely tuned over a very wide range by varying the fluorination degree of LDGMs, and further demonstrate that the thermal defluorination of F-LDGMs generates a high concentration of vacancies and facilitates superdoping.

### Performance of N-superdoped graphene

As reported, N-, S- or B-doping can change the physical and chemical properties of LDGMs and thus greatly widen their potential applications^7^,^8^,^9^,^10^,^11^,^12^,^13^,^14^,^15^,^16^,^17^,^18^,^19^,^20^,^21^,^22^,^23^,^24^,^25^,^26^,^27^,^28^,^29^,^30^,^31^,^32^,^33^,^34^,^35^. The successful superdoping of LDGMs with N, S or B offers an effective platform to study the fundamental properties and explore the applications of superdoped graphene, GQDs and SWCNTs. Graphene doped with high levels of N-6 and/or N-5 is highly desirable for supercapacitors, fuel cells and energy-storage devices, and as a ferromagnetic material^26^,^29^,^30^,^31^ and so on. As examples, we have investigated only supercapacitors, the ORR, magnetism and photoluminescence (PL).

**Figure 5:**
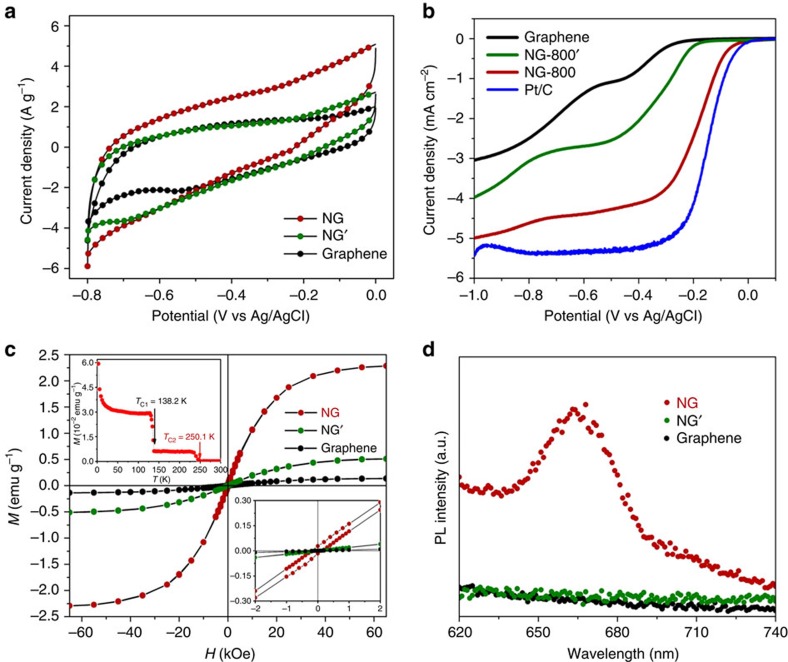
Performance of superdoped graphene with a high level of N. (**a**) Representative CV curves of graphene, NG' and NG in 6 M KOH solution at a scan rate of 5 mV s^−1^. (**b**) Linear sweep voltammetry (LSV) curves for graphene, NG-800', NG-800 and Pt/C on a rotating disk electrode (RDE; 1,600 r.p.m.) in 0.1 M KOH solution with a scan rate of 5 mV s^−1^. (**c**) Typical *M*–*H* curves of graphene, NG' and NG measured at 2 K. Insets are the *M*—*T* curve of NG measured from 2 to 300 K under an applied field *H*=500 Oe (top left of panel) and part of the magnetization curves (bottom right of panel). (**d**) PL spectra of graphene, NG' and NG excited at 500 nm.

Graphene materials have recently shown better performance in supercapacitor devices than conventional carbon electrodes, and the specific capacity of pristine graphene can be increased four times by N-doping with N-5 and/or N-6 (ref. 37). The reason for this is considered to be that N-doping can increase the binding energy of electrolyte ions at the graphene surface, resulting in N-doped surfaces being able to accommodate more ions and thus have a higher charge-storage capacity. The performance of our graphene and NG supercapacitors was characterized through galvanostatic charge–discharge and cyclic voltammetry (CV) measurements. From the CV curves (Fig. 5a), the corresponding specific capacitance (*C*_s_) was calculated of 353.8 F g^−1^, which is 66% higher than that of graphene (213.2 F g^−1^). The high *C*_s_ of NG can also be confirmed by the galvanostatic charge/discharge curves and the specific capacitances of the electrode at 5 and 10 A g^−1^ were calculated to be about 390 and 354 F g^−1^, respectively (Supplementary Fig. 9a). Our NG exhibits the best performance among all graphene-based supercapacitors ever reported^29^,^30^,^31^, demonstrating that a high N-doping level can greatly improve the specific capacitance of graphene. Furthermore, one finds that the CV curves are in the form of a triangle with little distortion, implying excellent capacitance behaviour and charge/discharge properties. CV curves of the NG electrode were also measured at different scan rates, ranging from 10 to 100 mV s^−1^ (Supplementary Fig. 9b). Near-rectangular CV curves without obvious redox peaks were observed, indicating the ideal capacitive behaviour of the supercapacitor. It is noteworthy that even at a high scan rate of 100 mV s^−1^, the shape of the CV curve remained close to rectangular and the specific capacitance retains 67.75% of its initial specific capacitance measured at 5 mV s^−1^. Moreover, it was found that the capacitance deterioration was less than 5.0% after 5,000 cycles (Supplementary Fig. 9c). All these results show that NG has a good rate capability and high capacity even under very fast charge transfer, suggesting its potential practical use in supercapacitors.

Pristine graphene lacks catalytic activity in the ORR and is not efficient in facilitating electron transfer^39^. However, as the doping of nitrogen in the graphite lattice can increase the electron density of states near the Fermi level, N-doping can improve the ORR activity of graphene, making it a promising metal-free electrocatalyst for the ORR^21^. It has been demonstrated that the high conductivity is favourable to the ORR activity of NG, and this can be achieved by annealing at 800 °C (refs 44, 45). We annealed FG in ammonia at temperatures from 400 to 800 °C and also found that 800 °C is the optimum temperature for ORR performance (Supplementary Fig. 10). Figure 5b shows the linear sweep voltammetry curves obtained for graphene and NG-800 for the same mass deposited on a glassy-carbon rotating disk electrode in an O_2_-saturated 0.1 M KOH aqueous solution at a scan rate of 5 mV s^−1^. Graphene shows an ORR onset potential at −0.25 V and has a cathodic current density of *ca*. −3.03 mA cm^−2^. After N-doping, the onset potential and the current density increase to −0.05 V and *ca*. −4.99 mA cm^−2^, respectively, close to those for Pt/C (−0.02 V and *ca*. −5.38 mA cm^−2^). It is clear that the onset potential of NG-800 is more positive than that of other N-doped carbon ORR catalysts reported^10^,^21^,^29^,^30^,^31^,^43^,^46^,^47^, indicating a more efficient ORR process on NG.

We have demonstrated that N-doping can increase the magnetization of graphene and induce ferromagnetism with a *T*_C_ at 100.2 K (ref. 26). We carried out magnetic measurements on graphene and NG, and found that at 300 K only pure diamagnetism can be observed in both materials (Supplementary Fig. 11a). At 2 K, graphene has pure spin 1/2 paramagnetism with a low magnetization of 0.14 emu g^−1^ (Fig. 5c and Supplementary Fig. 11b,c). Interestingly, NG shows clear ferromagnetism with an obvious coercive field of 190 Oe and a remanent magnetization of 0.028 emu g^−1^ (bottom right inset of Fig. 5c). Moreover, the magnetization of NG is high, up to 2.3 emu g^−1^ (Supplementary Fig. 11d), which is the highest recorded intrinsic magnetization for graphene derivatives. We performed an *M*–*T* measurement and found two clear *T*_C_'s at 138.2 and 250.1 K (top left inset of Fig. 5c). This can also be confirmed by the *M*–*H* curves measured at 100, 200 and 300 K (Supplementary Fig. 11e and Supplementary Note 1) and the zero-field cooling *M*–*T* curve (Supplementary Fig. 11f). Apparently, high-level N-doping results in a significant increase in the magnetization of graphene and the generation of near RT ferromagnetism. In addition, creating a PL peak in visible region in graphene is important for various applications. It is very interesting to find an obvious PL at 677 nm (Fig. 5d), indicating the clear creation of a PL peak in visible region by high level N-doping.

We have shown four examples of the excellent performance of NG such as the highest capacitance, superior ORR properties close to those of Pt/C, near-RT ferromagnetism with the highest magnetization and the clear creation of a PL peak in visible region. These properties are far superior to those reported for graphene or any other graphene derivatives. For comparison, we have investigated supercapacitors, magnetism and the PL of NG', and ORR of NG-800' obtained by direct annealing pristine graphene in ammonia. The conventional N-doped graphene materials show much lower performance regarding capacitance (Fig. 5a), ORR (Fig. 5b), magnetism (Fig. 5c) and PL (Fig. 5d) than those with very high N contents produced by superdoping. Clearly, all these results demonstrate that a high level of N-doping can significantly change the properties of graphene. Furthermore, the superdoping of graphene with N over an adjustable wide range will allow one to search for the optimum N-doping level for each property. Consequently, we examined the performance of NG with different N-doping levels and found that in capacitance (Supplementary Fig. 12a,b), magnetism (Supplementary Figs 13–16, Supplementary Table 7 and Supplementary Fig. 17) and PL (Supplementary Fig. 17), the higher the N-doping level, the better the performance. As a high annealing temperature is good for ORR activity, we prepared NG-800 samples with different N-doping levels by annealing FG samples with different fluorination degrees at 800 °C in ammonia (Supplementary Table 8). The results (Supplementary Fig. 18) showed that the higher the N-doping level, the better the performance.

## Discussion

We have reported a fluorination-followed-by-annealing method for the efficient doping of graphene, GQDs and SWCNTs with very high levels of N, S or B. The excellent performance of NG with a very high N-doping level in supercapacity, the ORR, magnetism and PL have proved the importance of the high doping level of heteroatoms in LDGMs. Moreover, we have illustrated that the doping level of N or S can be precisely adjusted over a very wide range, which demonstrates the high efficiency of this doping method and allows one to search for the optimum doping level for the performance optimization of graphene, GQDs and SWCNTs in many applications. We believe that the method may also be applicable to the superdoping of LDGMs with other heteroatoms such as P, Si and so on, in which vacancies are necessary for the doping, and to superdope LDGMs in other forms, such as thin films and single nanodevices, with high levels of N, S or B.

## Methods

### Graphene

Graphene was synthesized by annealing of graphene oxide (GO) in Ar at 700 °C (ref. 26). Herein, GO was prepared by Hummer's method. In a typical experiment, the mixture of 8 g graphite, 8 g NaNO_3_, 48 g KMnO_4_ and 384 ml condensed H_2_SO_4_ was stirred for 1.5 h at 0 °C, and then followed by another 2 h stirring at 35 °C. Thereafter, 320 ml H_2_O was added into the mixture within 15 min at a steady flow and the premixture of 800 ml H_2_O and 40 ml H_2_O_2_ was added within 6 min at a steady flow. During the process, the temperature of the solution was fixed at 35 °C. The obtained solution was washed for 13 times with deionized (DI) water by repeated 30-min centrifugation at 13,000 r.p.m. Next, the sediment was re-dispersed into DI water and centrifuged at 6,000 r.p.m. for 10 min and only the supernatant was left to get GO sheets with high few-layer ratio. To obtain highly pure GO sample without contamination, GO was washed with hydrochloric acid for 15 times and then with DI water for 10 times by repeated 30-min centrifugation at 13,000 r.p.m. Thereafter, GO powder was obtained after freeze drying. Finally, graphene was obtained followed by Ar annealing at 700 °C.

### Graphene quantum dots

GO quantum dots (GOQDs) were prepared from Vulcan XC-72 carbon black (Cabot Corporation, USA) by a modified Dong's method^48^. In a typical procedure, 1.6 g XC-72 carbon black was put into a 1,000-ml round bottom flask and 300 ml HNO_3_ (15 mol l^−1^) was added. Then, the mixture was stirred by magnetic stirrer and was heated in an oil bath at 135 °C for 24 h. After the mixture was heated in the oil bath at 180 °C for 10 h in Ar (60 ml min^−1^) to evaporate the concentrated nitric acid, a light black solid was obtained. The light black solid was redissolved in 2 l DI water followed by centrifuging (13,000 r.p.m.) for 30 min to remove the unoxidized carbon black or big particles and a supernatant was obtained. Subsequently, the obtained supernatant was diluted to 3.6 l solution with DI water and then the obtained solution was vacuum filtered through 220-nm microporous membrane to further remove the unoxidized carbon black or big particles. Next, the obtained solution was further filtered through 25-nm microporous membrane to remove big particles. Thereafter, the reddish-brown GOQDs solution was obtained. After rotary evaporation at 80 °C to remove the water of the solution, 150 ml concentrated GOQDs solution was obtained. The concentrated solution was dried by vacuum freeze drying and ∼1.1 g brown GOQDs powder was obtained. After annealing GOQDs powder in Ar at 700 °C for 1 h, GQDs were obtained.

### Single-wall carbon nanotubes

SWCNTs (XFS02, purity: >90%; outer diameter: <2 nm, length: 5–30 μm) were purchased from Nanjing XFNANO Materials Tech Co., Ltd (Nanjing, China).

### Fluorination of graphene

The FG samples (FG-1, FG-2, FG-3, FG-4, FG-5, FG-6 and FG) were obtained by annealing the mixture of graphene and XeF_2_ with different mass ratios in a Teflon container at 200 °C for 30 h in Ar.

### Fluorination of GQDs

The F-GQDs samples (F-GQDs-1, F-GQDs-2 and F-GQDs-3) were obtained by annealing the mixture of GQDs and XeF_2_ (Alfa Aesar, China) with different mass ratios in a Teflon container at 200 °C for 30 h in Ar^42^.

### Fluorination of SWCNTs

F-SWCNT-1, F-SWCNT-2 and F-SWCNT-3 were obtained by annealing the mixture of SWCNT and XeF_2_ with different mass ratios in a Teflon container at 200 °C for 30 h in Ar. F-SWCNT-4 and F-SWCNT were obtained by annealing the mixture of F-SWCNT-3 and XeF_2_ for 30 h in Teflon container in Ar at 220 °C and 240 °C, respectively.

### N-doping of FG and graphene

The NG samples (NG-1, NG-2, NG-3, NG-4, NG-5, NG-6 and NG) or NG' were prepared by annealing the FG samples (FG-1, FG-2, FG-3, FG-4, FG-5, FG-6 and FG) or pristine graphene in ammonia at 500 °C for 1 h under ambient pressure. Herein, the numeric number is the serial number of the FG samples with different fluorination degree. Using the serial number of the FG samples, which correspond to NG samples (NG-1, NG-2, NG-3, NG-4, NG-5 and NG-6) with different N-doping level, are capable of being put into a one-to-one relationship.

The NG samples (NG-400, NG-500 (*viz*. NG), NG-600, NG-700 and NG-800, numeric numbers denote the annealing temperature) were prepared by annealing FG in ammonia at different temperatures for 1 h under ambient pressure. In short, the FG samples were spread on a quartz boat that was placed in a quartz tube reactor (1.0 m in length) in a tubular furnace (0.4 m in length). The quartz tube reactor is movable with protective atmosphere and thus the quartz boat can be introduced into and pulled out from the tubular furnace by simply moving the quartz tube reactor. Next, ammonia (99.99%) at a rate of 100 s.c.c.m. was maintained for 30 min, to get rid of air. After heating the furnace from RT to 500 °C, the quartz boat was introduced into the furnace, while ammonia was kept at a rate of 20 s.c.c.m. After 1 h, the tube was rapidly moved out and the NG samples were obtained.

The NG-800 samples (NG-800-1, NG-800-2, NG-800-3 and NG-800) or NG-800' were prepared by annealing the FG samples (FG-1, FG-3, FG-5 and FG) or graphene in ammonia at 800 °C for 1 h under ambient pressure.

NG” was prepared by two steps. First, FG was annealed in Ar at 500 °C for 0.5 h, after that, it was annealed in ammonia at 500 °C for 1 h. In short, Ar (99.99%) at a rate of 100 s.c.c.m. was maintained for 20 min, to get rid of air; after annealing the furnace from RT to 500 °C, FG were introduced into the reaction tube with Ar rate of 20 s.c.c.m. for 0.5 h at atmospheric pressure. Second, the ammonia was introduced into the reaction tube with the rate of 20 s.c.c.m. for 1 h. After the furnace cooled to RT, NG” was obtained.

### N-doping of F-GQDs and GQDs

The N-GQDs samples (N-GQDs-1, N-GQDs-2, N-GQDs-3 and N-GQDs) or N-GQDs' were prepared by annealing the F-GQDs samples (F-GQDs-1, F-GQDs-2, F-GQDs-3 and F-GQDs) or GQDs in ammonia at 500 °C for 1 h under ambient pressure. In short, the F-GQDs samples or GQDs were was spread on a quartz boat, which was placed in a quartz reaction tube in a chemical vapour deposition furnace, and then ammonia (99.99%) at a rate of 100 s.c.c.m. was maintained for 30 min, to get rid of air. After heating the furnace from RT to 500 °C, the quartz boat was introduced into the furnace, while ammonia was kept at a rate of 20 s.c.c.m. After 1 h, the tube was rapidly moved out and the N-GQDs samples were obtained.

### N-doping of F-SWCNTs and SWCNTs

The N-SWCNTs samples (N-SWCNTs-1, N-SWCNTs-2, N-SWCNTs-3, N-SWCNTs-4 and N-SWCNTs) or N-SWCNTs' were prepared by annealing the F-SWCNTs samples (F-SWCNTs-1, F-SWCNTs-2, F-SWCNTs-3, F-SWCNTs-4 and F-SWCNTs) or SWCNTs in ammonia at 500 °C for 1 h under ambient pressure.

### S-doping of F-LDGMs and LDGMs

S-doped LDGMs (S-LDGMs) were prepared by annealing F-LDGMs or LDGMs in sulfur gas carried by Ar. The sulfur powder (Mat-cn, China) was placed in a quartz boat that was placed in a quartz reaction tube in a tubular furnace at Ar inlet and F-LDGMs or LDGMs were put in another quartz boat at the outlet. Before heating, the system was flushed by Ar at a rate of 100 s.c.c.m. for 30 min. Then, under a constant Ar flow rate of (10 s.c.c.m.), the furnace was heated to 400 °C and held at this temperature for 10 min. After that, the furnace was cooled to RT and S-LDGMs were obtained.

### B-doping of FG and graphene

BG and BG' were respectively prepared by annealing FG and graphene in a gas mixture of BCl_3_ (∼99.99%) and Ar (1:4 v/v) with a total flow rate of 100 ml min^−1^ at 800 °C for 1 h under ambient pressure^9^. FG or graphene was spread on a quartz boat that was placed in a quartz reaction tube in a chemical vapour deposition furnace and then Ar at a flow rate of 100 s.c.c.m. was maintained for 30 min, to get rid of the air. After heating the furnace from RT to 800 °C, the quartz boat was introduced into the furnace, while the gas mixture flow of BCl_3_ and Ar was maintained at 100 s.c.c.m. One hour later, the tube was rapidly moved out from the furnace in Ar and BG or BG' were obtained.

For details about the masses of pristine, fluorinated and N-LDGMs, S-LDGMs or B-doped LDGMs used in the synthesis, see Supplementary Tables 9–15.

### Microstructure characterization

The structure was investigated by a TEM (model JEM–2100, Japan). The sizes of GQDs were calculated by Adobe Photoshop software (version CS6). XPS measurements were performed on a PHI5000 VersaProbe (ULVAC-PHI, Japan). XPS was performed using 200 W monochromated Al Kα radiation. A 500-μm X-ray spot was used for XPS analysis. Typically, the hydrocarbon C 1 s line at 284.8 eV from adventitious carbon was used as an energy reference. The XPS peak fitting programme XPSPEAK 4.1 was used for the spectra processing. Raman spectra were obtained by an Raman system (Renishaw, England) using a 514.5-nm laser as the light source. Elemental mapping images were recorded in an image aberration-corrected TEM (FEI TITAN 80-300 operating at 80 kV) using the energy-filtered TEM mode.

### Electrochemical measurements

The electrochemical properties of the as-obtained products were investigated using a three-electrode cell configuration at RT. The working electrodes were fabricated by mixing the prepared powders with 15 wt% acetylene black and 15 wt% polytetrafluorene-ethylene binder. A small amount of *N*-methyl-2-pyrrolidone was added to the mixture, to produce a homogeneous paste. Next, the mixture was pressed onto nickel foam current collectors (1.0 cm × 1.0 cm) to make electrodes. Platinum foil and Ag/AgCl were used as the counter and reference electrodes, respectively. Before the electrochemical test, the prepared electrode was soaked overnight in a 6-M KOH solution. Electrochemical characterization was carried out in a conventional three-electrode cell with a 6-M KOH aqueous solution as the electrolyte. CV and galvanostatic charge–discharge measurements were conducted on an electrochemical workstation (CHI 660D, CH Instrument, USA). From the CV curves, the corresponding specific capacitance (*C*_s_) can be calculated based on the following equation


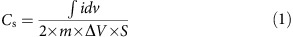


where *∫idv* is the integrated area under the CV curve, *m* is the mass of the electrode active material in grams, Δ*V* is the scanned potential window in volts and *S* is the scan rate in volts per second. From the galvanostatic charge–discharge curve, the specific capacitance can be calculated from *C*_s_=*I*Δ*t*/Δ*Vm*, where *I* is the discharge current, Δ*t* is the total discharge time, *m* is the mass of active material and Δ*V* is the potential difference in the discharge process^49^.

### ORR measurements

ORR measurements were performed on a computer-controlled potentiostat (CHI 760E, CH Instrument, USA) with a three-electrode cell equipped with gas flow system. A Pt wire and a Ag/AgCl electrode filled with saturated KCl aqueous solution were used as the counter electrode and reference electrode, respectively. A 0.1-M KOH solution was prepared as the electrolyte and saturated with oxygen by bubbling oxygen gas through it for 30 min before measuring ORR activities. To prepare the NG-loaded working electrode, NG was dispersed in a mixture of water and isopropanol (4:1 v/v) containing 0.05 wt.% Nafion. The electrocatalytic activities of NG for the ORR were measured using a rotating disk electrode (Pine Instruments, MSR analytical rotator, USA) with a scan rate of 5 mV s^−1^. NG dispersion (10 μl of 2 mg ml^−1^) was transferred onto the GC electrode (5 mm diameter, 0.196 cm^2^ geometric area).

### Magnetic measurements

The magnetic properties of the powdered samples were measured using a superconducting quantum interference device magnetometer with a sensitivity better than 10^–8^ emu (Quantum Design MPMS–XL, USA) and all data were corrected for the diamagnetic contribution by subtracting the corresponding linear diamagnetic background measured at 300 K. The concentrations of magnetic impurity elements (such as Fe, Co, Ni or Mn) of all the samples are below 40 p.p.m. (Supplementary Table 16) measured by ICP spectrometry (Jarrell–Ash, USA). The magnetization at 2 K is composed of two parts: paramagnetization (PM) and ferromagnetization, which can be expressed as *M*_total_=*M*_para_+*M*_ferro_. Considering the fact that ferromagnetic mass magnetization can saturate at a high applied field approximately, one can fit the PM at a high applied field. The PM is fitted to the Brillouin function


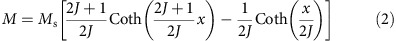


where *x*=*gJμ*_*B*_*H*/(*k*_*B*_*T*), *M*_s_=*NgJμ*_*B*_, *k*_*B*_ the Boltzmann constant, *N* the number of magnetic moments present, *J* the angular momentum number and *g* is the Landau factor. By subtracting the paramagnetic signal from the observed data, one can obtain the remaining ferromagnetic moment. From the saturated paramagnetic magnetization added to the saturated ferromagnetic magnetization, the *M*_s_ of the sample can be calculated.

### PL measurements

The PL spectra were measured at ambient conditions by a spectrofluorophotometer (Shimadzu RF-5301PC, Japan) using a Xe lamp as the light source. For PL spectra investigation, ∼1 mg of powdered sample was ultrasonically dispersed in 20 ml ethanol for 1 h. After that, the solution was used.

## Additional information

**How to cite this article:** Liu, Y. *et al*. Elemental superdoping of graphene and carbon nanotubes. *Nat. Commun.* 7:10921 doi: 10.1038/ncomms10921 (2016).

## Supplementary Material

Supplementary Information Supplementary Figures 1-18, Supplementary Tables 1-16, Supplementary Notes 1-2 and Supplementary References
