# Supplementary material for: Elemental superdoping of graphene and carbon nanotubes
Source: Nat Commun. 2016 Mar 4;7:10921. doi: 10.1038/ncomms10921 (PMC4785233; doi:10.1038/ncomms10921)
Supplement: Supplementary Information — Supplementary Figures 1-18, Supplementary Tables 1-16, Supplementary Notes 1-2 and Supplementary References [file ncomms10921-s1.pdf]

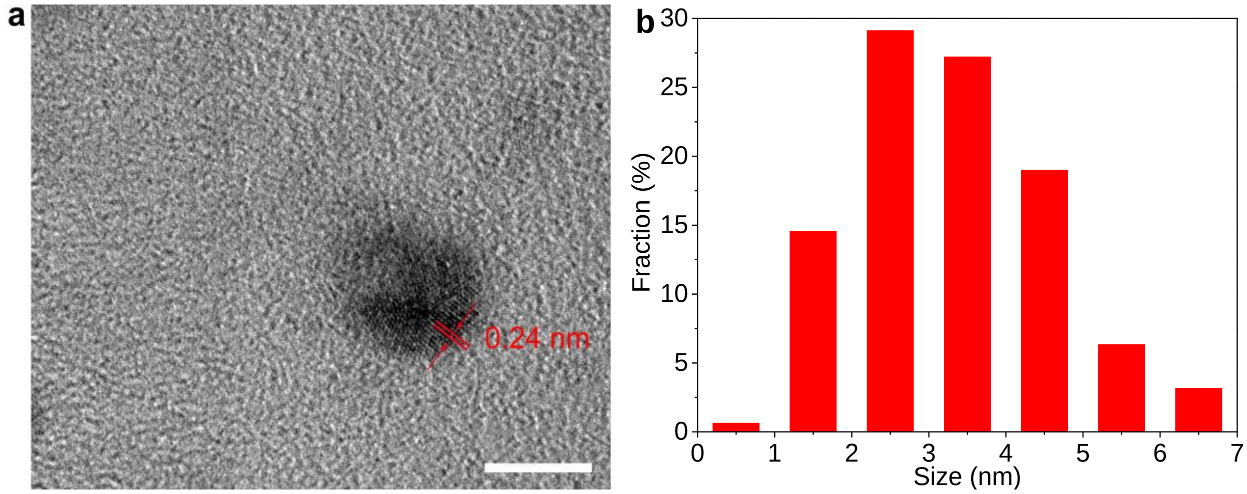

Supplementary Figure 1 | Microstructures of GQDs. (**a**) HRTEM image. Scale bar, 5 nm. (**b**) Diameter distribution. The HRTEM image indicates a high crystallinity of the GQDs, where lattice fringes with an in-plane (0-110) lattice spacing of 0.24 nm can be seen. Supplementary Fig. 1b shows the diameter distribution of GQDs counted from Fig. 1a in the main text, it is found that they are mainly distributed in the range of 1-7 nm.

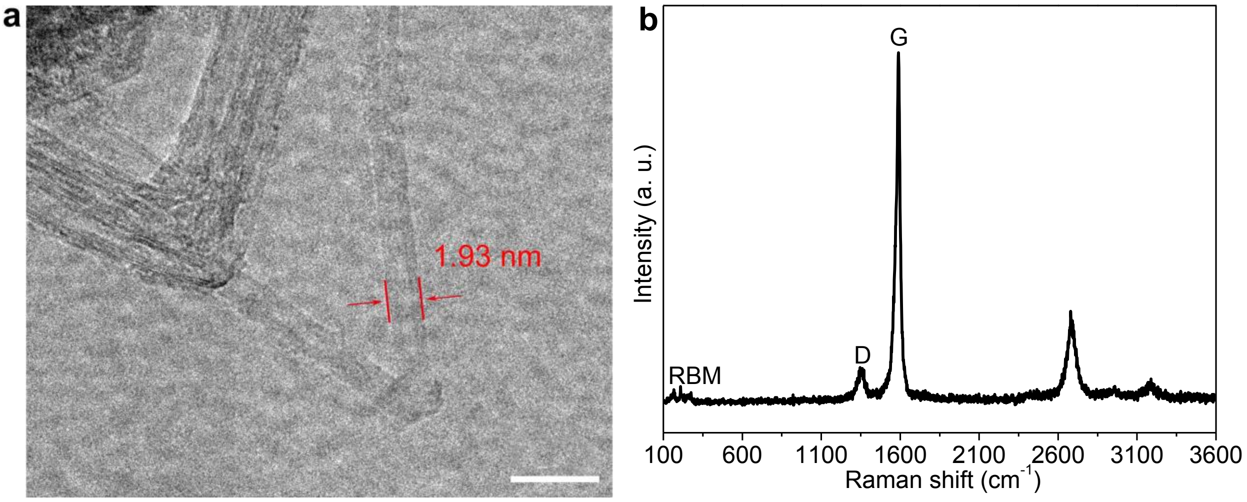

Supplementary Figure 2 | Microstructures of SWCNTs. (**a**) HRTEM image. Scale bar, 5 nm. (**b**) Raman spectrum**.** The diameter of the SWCNTs can be approximated by the simple equation $\omega_{RBM} = A/d_t + B$, where d_t is the CNT diameter, $A = 217.8 \times 10^{-7}$, and $B = 15.7\ (cm^{-1})$[1]. Hence the RBM mode positions of the most intense bands (ca. 200 cm^{-1}) correspond to tube diameters of ca. 1.2 nm.

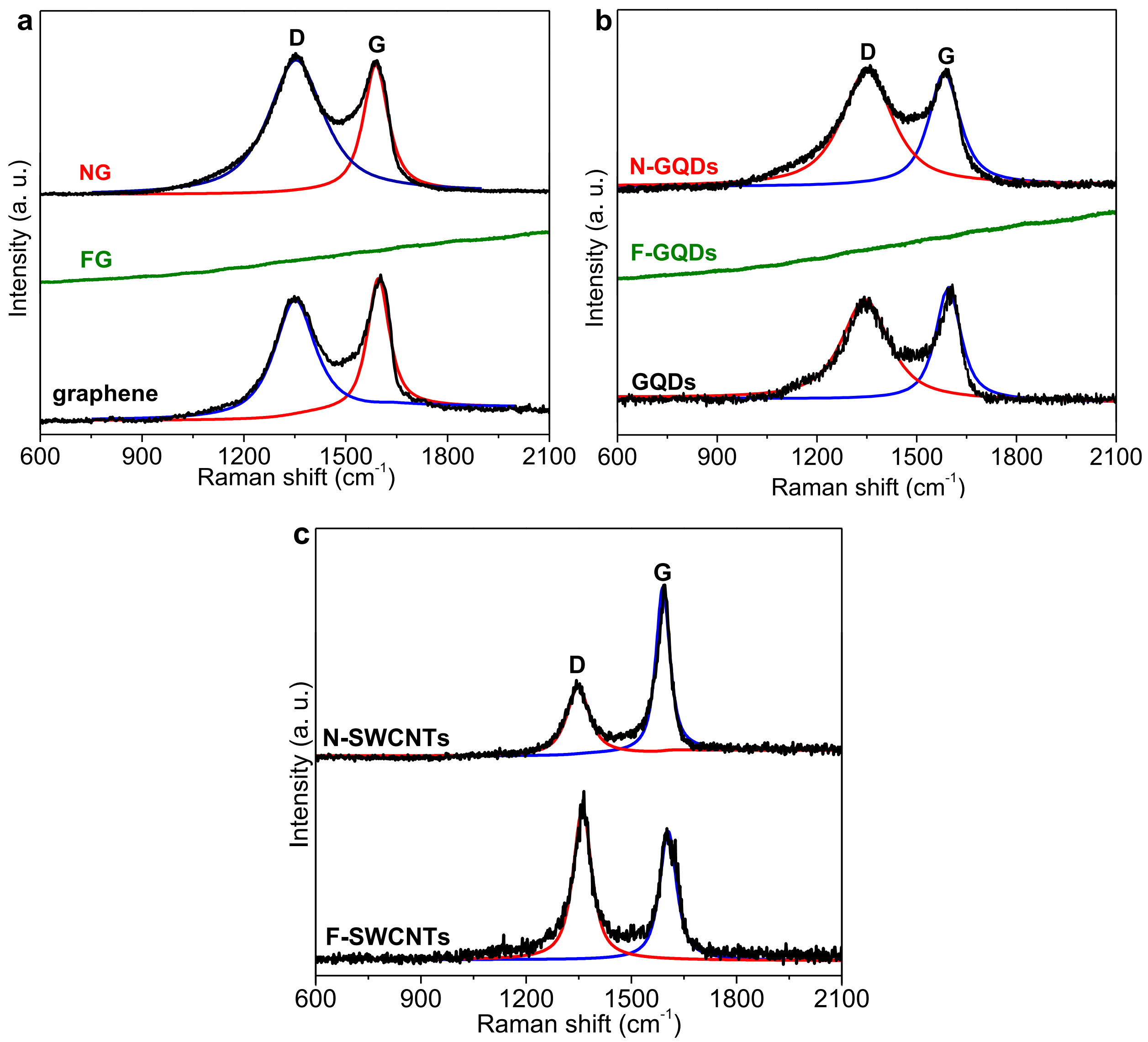

Supplementary Figure 3 | Raman spectra of the pristine, fluorinated, and N-doped LDGMs. Raman spectra of (**a**) graphene, FG, and NG, (**b**) GQDs, F-GQDs, and N-GQDs and (**c**) F-SWCNTs and N-SWCNTs. As shown, LDGMs show a G peak at ca. 1588 cm^{-1} and a D peak at ca. 1348 cm^{-1}, and the intensity ratios of the D band to G the band (I_D/I_G) of GQDs, SWCNT and graphene are 1.67, 0.10 and 1.64, respectively. After fluorination, both D and G peak of F-GQDs and FG disappeared, suggesting that F-GQDs and FG are approximately fully fluorinated[2]. Besides, I_D/I_G of F-SWCNTs increases to 1.12, suggesting that it is partially fluorinated. After ammonia annealing, both the D and G peaks of N-GQDs and NG appeared, and I_D/I_G of

N-SWCNTs decreased. Compared to GQDs, SWCNT and graphene, N-GQDs, N-SWCNTs and NG show the higher I_D/I_G of ca. 1.68, 0.66 and 2.34, respectively, suggesting the high-level N-doping[3].

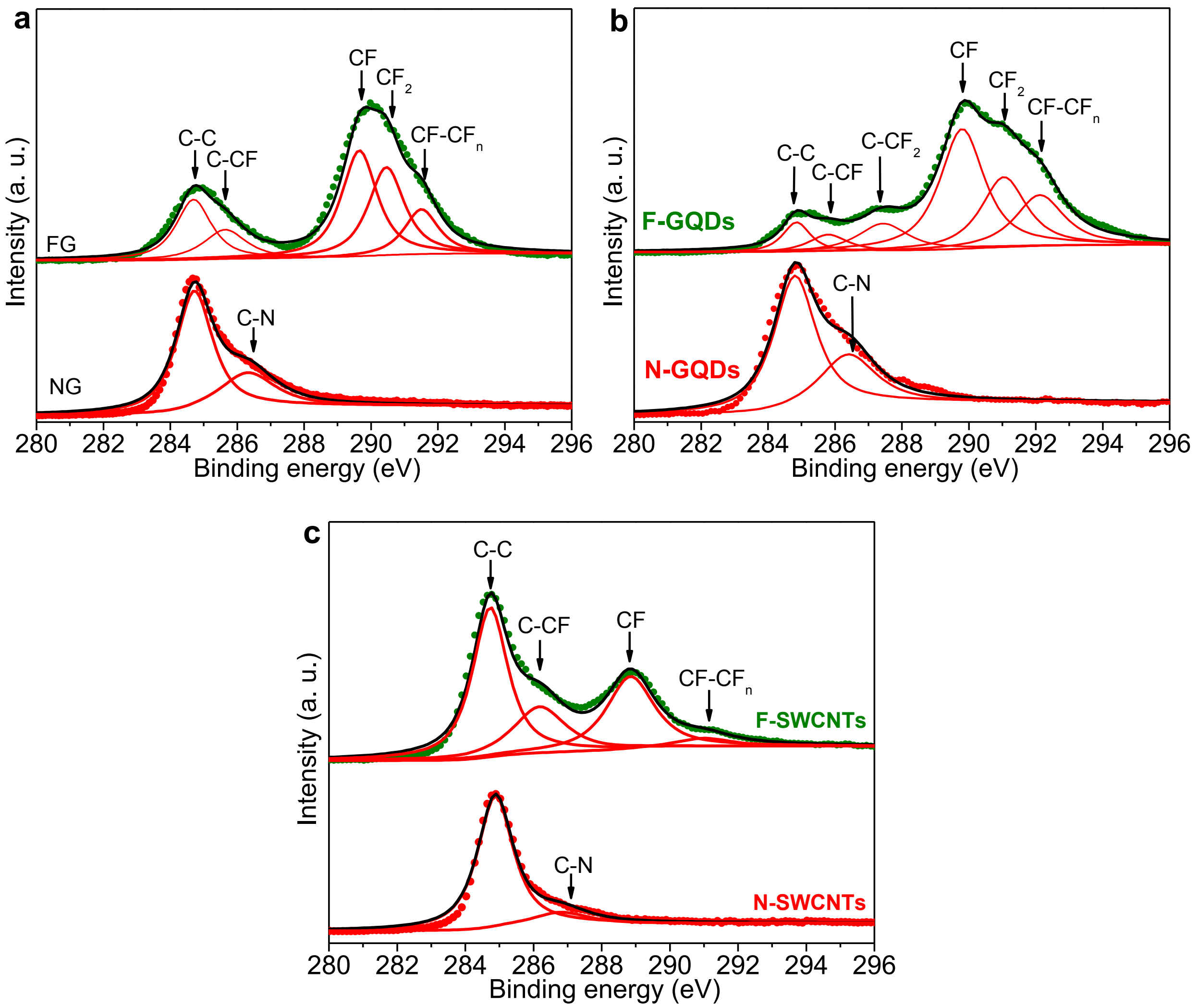

Supplementary Figure 4 | XPS spectra of fluorinated and N-doped LDGMs. The high-resolution C 1s XPS spectra of (**a**) FG and NG**,** (**b**) F-GQDs and N-GQDs and (**c**) F-SWCNTs and N-SWCNTs. The C 1s XPS spectra of F-LDGMs show a peak at 284.8 eV assigned to C–C sp^2 bonds. The peaks corresponding to C–CF, C–CF_2, CF, CF_2, and CF–CF_n bonds can be clearly observed. After N-doping, the C 1s XPS spectra of N-LDGMs show that the CF bonds disappear and only a peak corresponding to C-N bond can be observed[4].

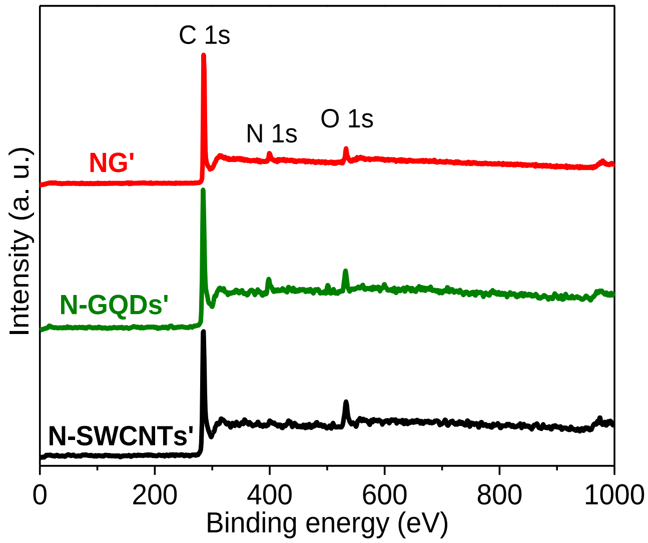

Supplementary Figure 5 | XPS spectra of NG', N-GQDs' and N-SWCNT' over a wide range of binding energies.

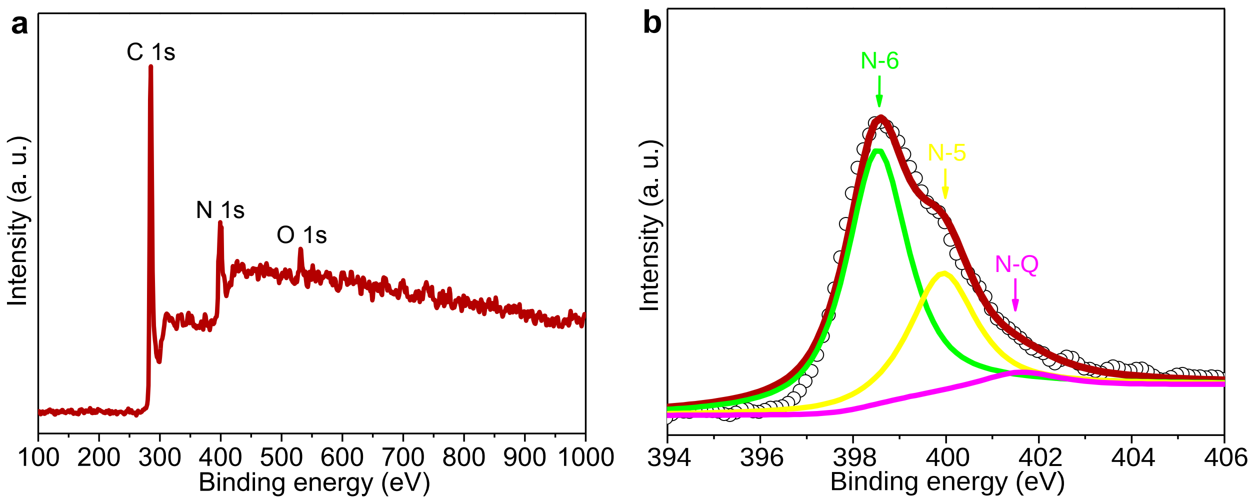

Supplementary Figure 6 | XPS spectra of NG''. (**a**) XPS spectrum over a wide range of binding energies. (**b**) The high-resolution N 1s XPS spectrum. The black circles and colorful lines are measured dots and fitting curves. The high-resolution N 1s XPS spectrum was deconvoluted into three subpeaks. The three subpeaks located at 398.3, 400.0 and 401.4 eV can be attributed to pyridinic N (N-6), pyrrolic N (N-5) and graphitic N (N-Q)[5], respectively. Based on the XPS results, the N-doping levels defined as N/C × 100 at% of total N and three N types of NG'' can be quantitatively obtained, which are shown in Supplementary Table 3.

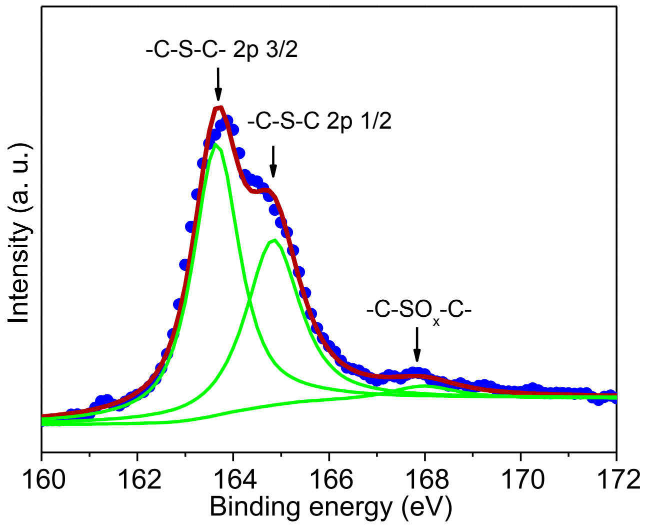

Supplementary Figure 7 | The high-resolution S 2p XPS spectrum of SG. The dots are measured data and the solid lines are fitting curves. As shown, SG contains sulfur atoms that are doped into the graphitic lattice, forming N-6 and N-5 like structures with neighboring carbon atoms[6].

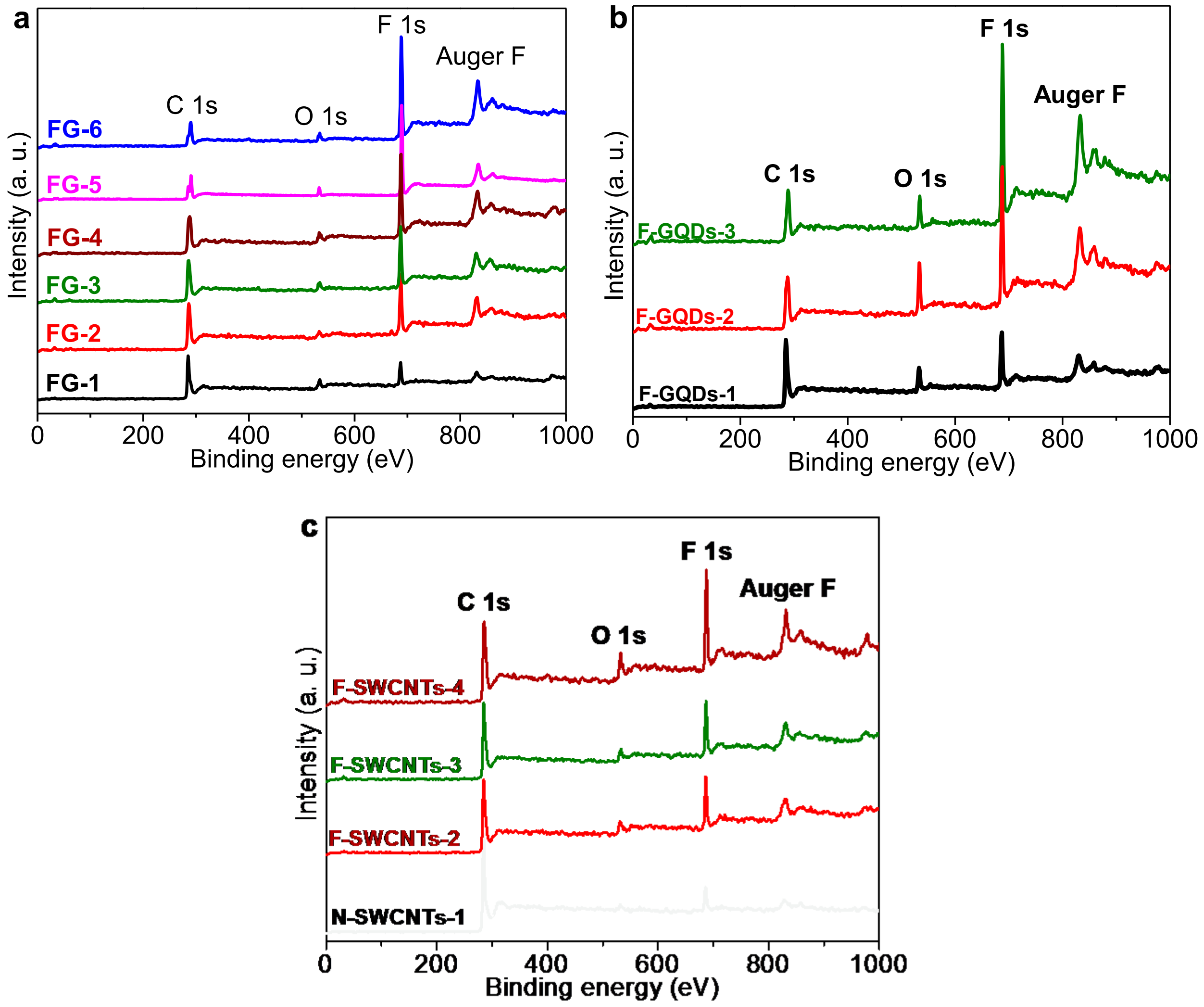

Supplementary Figure 8 | XPS spectra of F-LDGMs. XPS spectra over a wide range of binding energies of (**a**) FG, (**b**) F-GQDs and (**c**) F-SWCNTs.

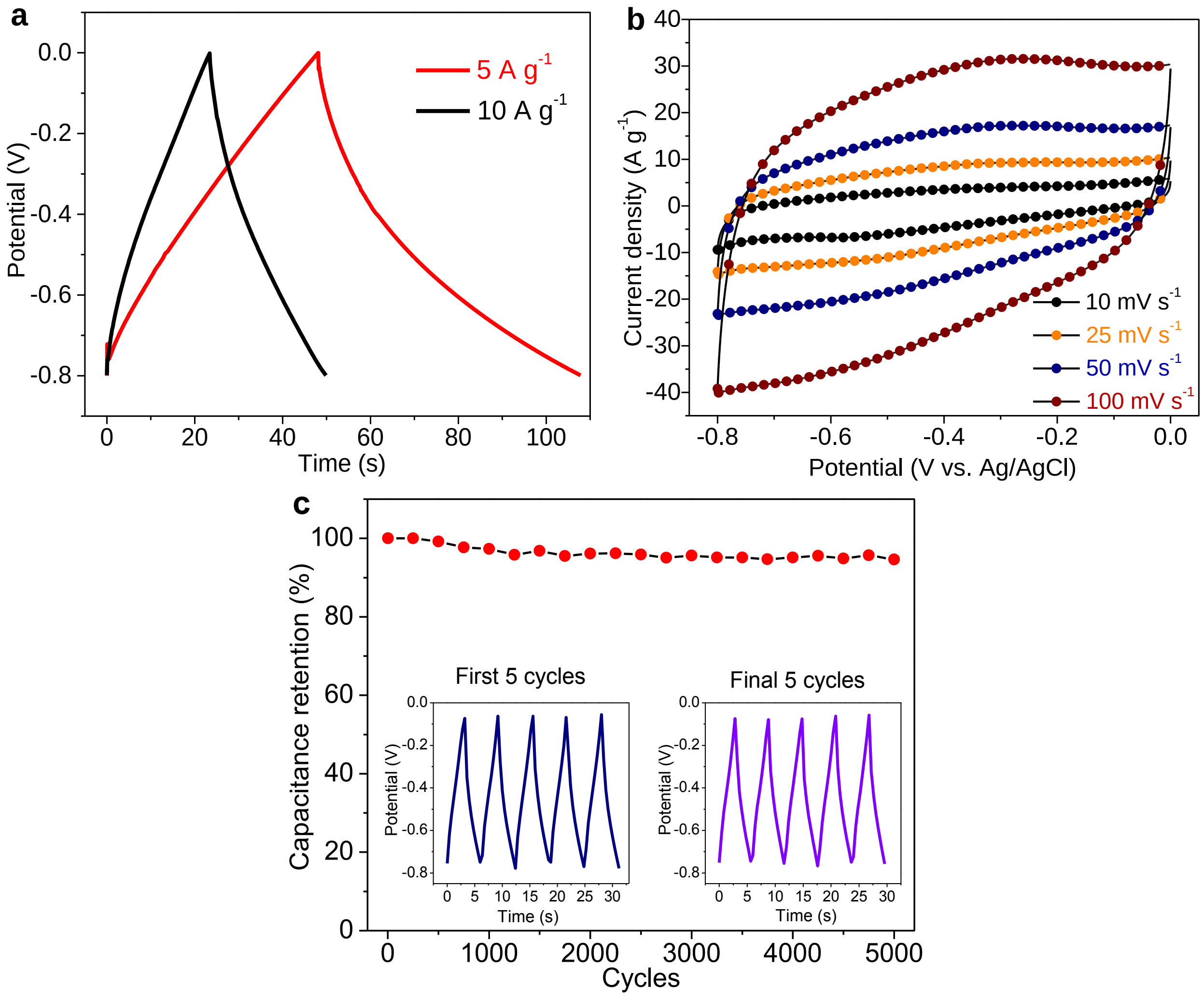

Supplementary Figure 9 | Electrochemical properties of NG-based device. (**a**) Galvanostatic charging/discharging curves for NG at current densities of 5 and 10 A g^{-1}. (**b**) Cyclic voltammograms (CV curves) of NG supercapacitor obtained in 6 M KOH solution at different scan rates. (**c**) Cycling behavior of the NG at the current density of 50 A g^{-1} between 0 and -0.8 V. The inset shows the galvanostatic charge/discharge curve for the sample in the first and final five cycles. As shown in Supplementary Figs. 9a,b, the C_s of NG electrode at 5 A g^{-1} and 10 A g^{-1} were calculated to be ca. 390 and 354 F g^{-1}, and the C_s at scan rate of 10-100 mV s^{-1} is 348.3, 303.4, 271.7 and 239.7 F g^{-1}, respectively. Moreover, one can find that (i) the capacity deterioration is less than 5.0% after 5000 cycles; and (ii) the shape of charge/discharge curve of the last 5 cycles is

almost similar to that of the first 5 cycles (Supplementary Fig. 9c), implying the high reversibility and long-term electrochemical stability of the NG electrode.

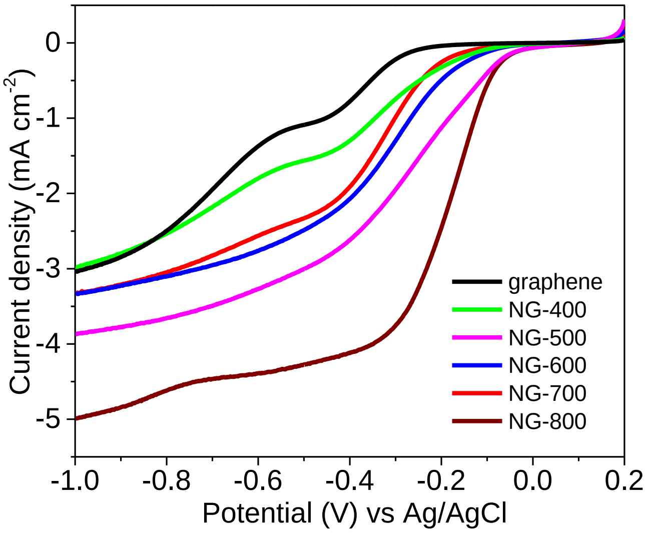

Supplementary Figure 10 | LSV curves for graphene and the NG samples obtained at different annealing temperatures in 0.1 M KOH solution with a scan rate of 5 mV s^{-1}. It is found that NG-800 has the highest current density and onset potential, suggesting that the optimal temperature to prepare NG for oxygen reduction reaction (ORR) activity is 800 ^{o}C**.**

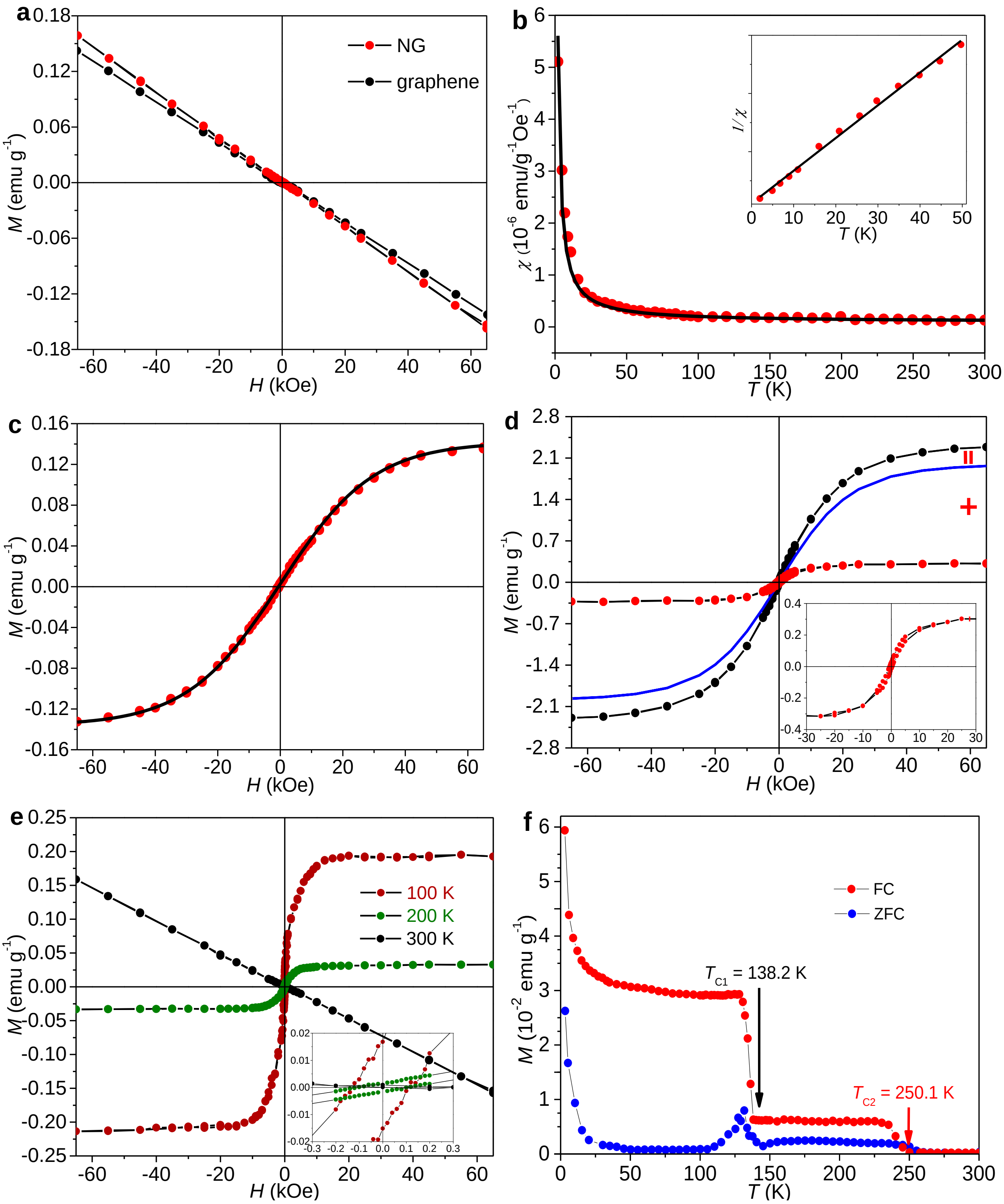

Supplementary Figure 11 | Magnetic properties of graphene and NG. (**a**) Mass magnetization ($M - H$) curve of graphene and NG measured by SQUID at 300 K. (**b**) Typical $\chi - T$ curve of graphene measured from 2 to 300 K under the applied field H = 3 kOe. Inset is the corresponding $1/\chi - T$ curve. Red symbols are the measurements and black solid line is fitted by the Curie law. (**c**)

M – *H* curve of graphene measured at 2 K. Red symbols are the measurements and black solid curve is fit to the Brillouin function. (**d**) Analysis of the 2 K *M* – *H* curve of NG. Black curve is the measured curve, and blue line is the fitting curve for paramagnetism by the Brillouin function with g = 2 and *J* = 1. Red curve is the ferromagnetic *M* – *H* curve by subtracting the paramagnetic *M* – *H* curve from the measured curve. Inset is a part of the ferromagnetic *M* – *H* curve. (**e**) *M* – *H* curves of NG measured at 100, 200, and 300 K, respectively. Inset is part of the *M* – *H* curves. (**f**) Field cooling (FC) and zero filed cooling (ZFC) *M* – *T* curves of NG under an applied field *H* = 500 Oe.

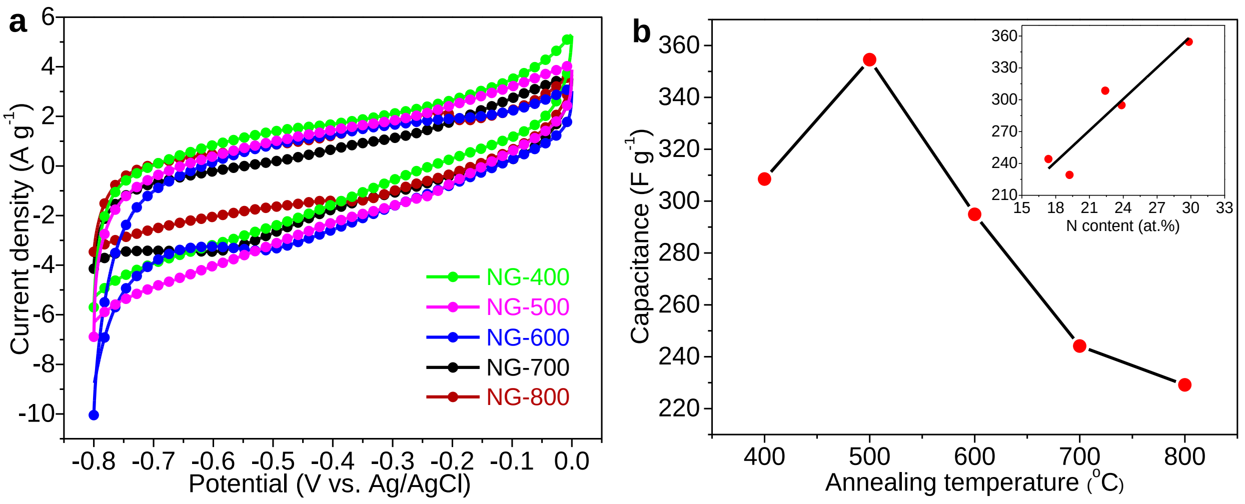

Supplementary Figure 12 | Electrochemical properties of the NG-based devices using the NG samples obtained at different annealing temperatures. (**a**) CV curves in 6 M KOH solution. (**b**) Dependence of specific capacitance on the annealing temperature. Inset is the dependence of specific capacitance on the N-doping level. The C_s of the NG samples obtained at different temperatures (NG-400, NG-500, NG-600, NG-700, and NG-800) at scan rate of 5 mV s^{-1} is 308.49, 354, 294.87, 244.10 and 229.14 F g^{-1}, respectively, suggesting that C_s is dependent on the N level.

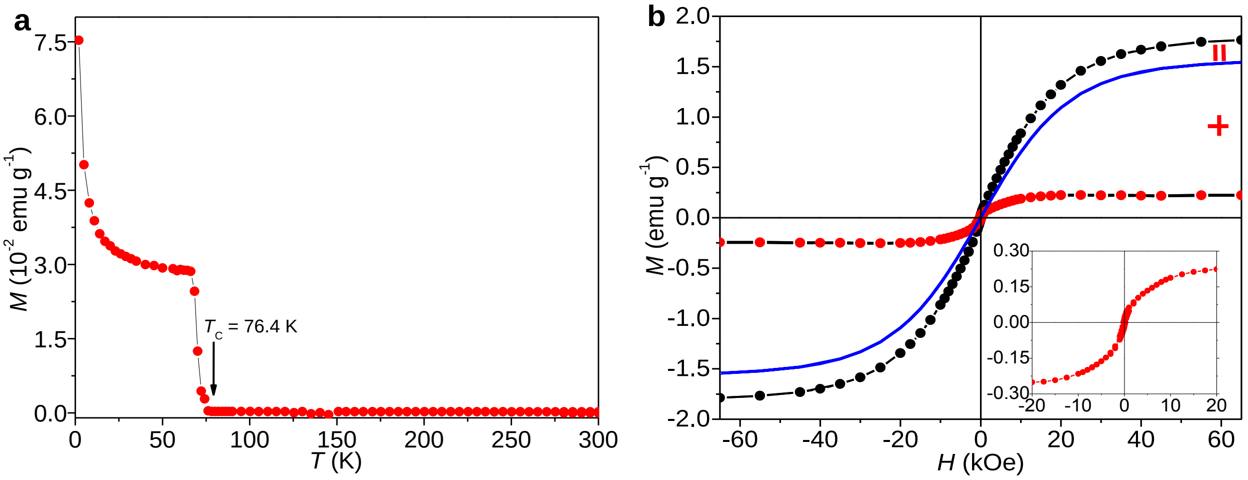

Supplementary Figure 13 | Magnetic properties of NG-400. (**a**) *M*–*T* curve measured from 2 to 300 K under the applied field *H* = 500 Oe. (**b**) Analysis of the 2 K *M* – *H* curve. Black curve is the measured curve, and blue line is the fitting curve for paramagnetism by the Brillouin function with *g* = 2 and *J* = 1. Red curve is the ferromagnetic *M*–*H* curve by subtracting the paramagnetic *M*–*H* curve from the measured curve. Inset is a part of the ferromagnetic *M* – *H* curve.

As shown in Supplementary Fig. 13a, it is found that T_C of NG-400 is ca. 76.4 K. The saturated paramagnetic magnetization is fitted to be 1.58 emu g^{-1}, and the saturated ferromagnetic magnetization is ca. 0.23 emu g^{-1} (Supplementary Fig. 13b). From saturated paramagnetic magnetization added with saturated ferromagnetic magnetization, one can calculate the M_s of NG is 1.81 emu g^{-1}.

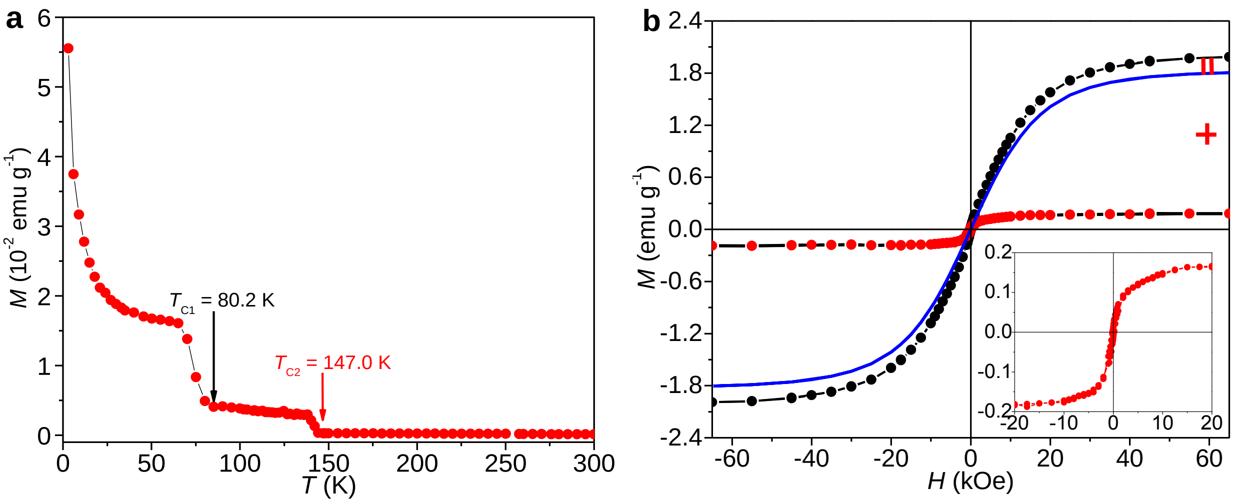

Supplementary Figure 14 | Magnetic properties of NG-600. (**a**) $M – T$ curve measured from 2 to 300 K under the applied field H = 500 Oe. (**b**) Analysis of the 2 K $M – H$ curve. Black curve is the measured curve, and blue line is the fitting curve for paramagnetism by the Brillouin function with g = 2 and J = 1.5. Red curve is the ferromagnetic $M – H$ curve by subtracting the paramagnetic $M – H$ curve from the measured curve. Inset is a part of the ferromagnetic $M – H$ curve.

It is found that T_C of NG-600 is ca. 80.2 and 147.0 K (Supplementary Fig. 14a). The saturated paramagnetic magnetization is fitted to be 1.82 emu g^{-1}, and the saturated ferromagnetic magnetization is ca. 0.18 emu g^{-1} (Supplementary Fig. 14b). From saturated paramagnetic magnetization added with saturated ferromagnetic magnetization, one can calculate the M_s of NG is 2.00 emu g^{-1}.

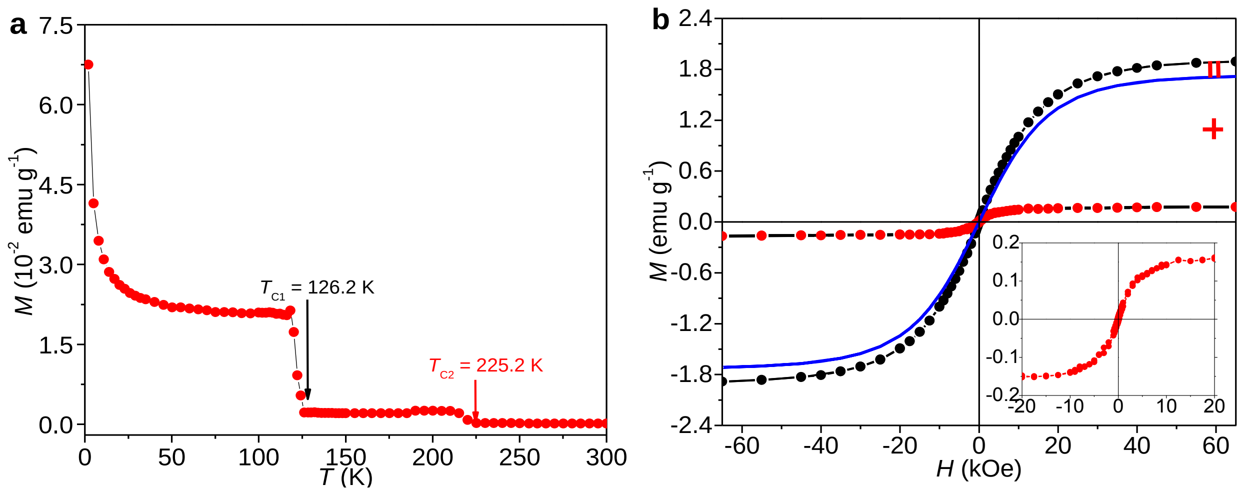

Supplementary Figure 15 | Magnetic properties of NG-700. (**a**) $M - T$ curve measured from 2 to 300 K under the applied field H = 500 Oe. (**b**) Analysis of the 2 K $M - H$ curve. Black curve is the measured curve, and blue line is the fitting curve for paramagnetism by the Brillouin function with g = 2 and J = 1.5. Red curve is the ferromagnetic $M - H$ curve by subtracting the paramagnetic $M - H$ curve from the measured curve. Inset is a part of the ferromagnetic $M - H$ curve.

As shown in Supplementary Fig. 15a, it is found that T_C of NG-700 is ca. 126.2 and 225.2 K. The saturated paramagnetic signal is fitted to be 1.72 emu g^{-1}, and the saturated magnetization of ferromagnetic signal is ca. 0.17 emu g^{-1} (Supplementary Fig. 15b). From saturated paramagnetic magnetization added with saturated ferromagnetic magnetization, one can calculate the M_s of NG is 1.89 emu g^{-1}.

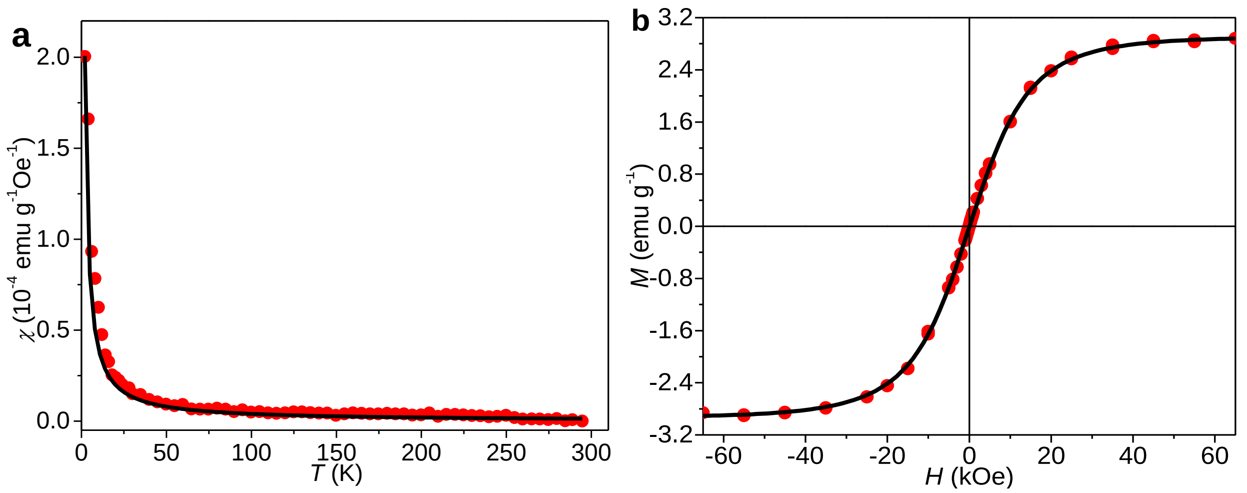

Supplementary Figure 16 | Magnetic properties of NG-800. (**a**) Typical $\chi - T$ curve measured from 2 to 300 K under the applied field H = 3 kOe. (**b**) $M - H$ curve measured at 2 K. Red symbols are the measurements and black solid curve is fit to the Brillouin function. It is found that NG-800 shows a typical paramagnetic behavior similar as graphene (Supplementary Fig. 16a). As shown by the fitting curve (Supplementary Fig. 16b), the Brillouin function provides good fits with g = 2 and J = 2, and M_s can be obtained which is 2.98 emu g^{-1}.

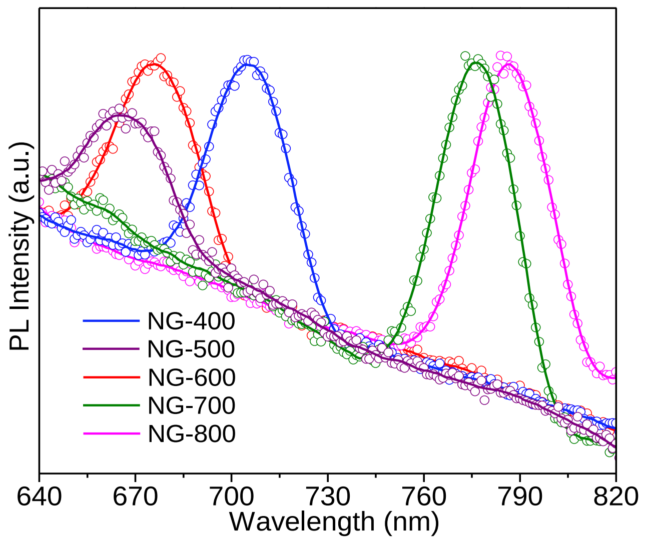

Supplementary Figure 17 | Photoluminescence (PL) spectra of the NG samples obtained at different annealing temperatures excited at 500 nm. The PL peak shifts with annealing temperature from 665 to 787 nm, revealing a formation of a bandgap from 1.57 to 1.86 eV. Approximately, it suggests that the bandgap of NG can be tuned by changing the N-doping level.

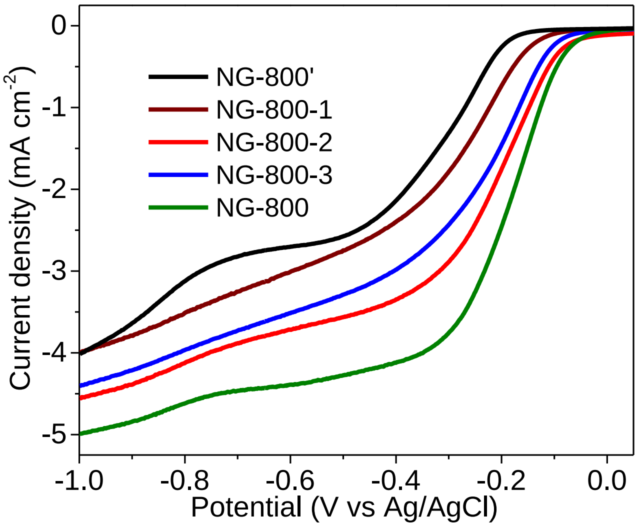

Supplementary Figure 18 | LSV curves for the NG-800 samples obtained by annealing FG with different fluorination degrees in 0.1 M KOH solution with a scan rate of 5 mV s^{-1}.

Supplementary Table 1 | The doping levels of total N, N-6, N-5 and N-Q of the NG samples obtained at different annealing temperatures, the numeric numbers denote the annealing temperatures.

Samples	N/C (at%)	N-6/C (at%)	N-5/C (at%)	N-Q/C (at%)
NG-400	22.40	12.77	8.51	1.12
NG-500	29.82	18.49	9.54	1.79
NG-600	23.86	15.51	6.68	1.67
NG-700	17.36	11.98	3.65	1.74
NG-800	19.24	12.70	3.46	3.08

Supplementary Table 2 | The doping levels of total N, N-6, N-5 and N-Q of the N-LDGMs. 'ND' denotes 'not found'.

Samples	N/C (at%)	N-6/C (at%)	N-5/C (at%)	N-Q/C (at%)
NG	29.82	18.49	9.54	1.79
N-GQDs	36.38	19.92	16.46	ND
N-SWCNTs	7.79	3.96	3.11	0.72

Supplementary Table 3 | The doping levels of total N, N-6, N-5 and N-Q of NG''.

Samples	N/C (at%)	N-6/C (at%)	N-5/C (at%)	N-Q/C (at%)
NG''	21.03	13.23	6.61	1.19

Supplementary Table 4 | The doping levels of total N, N-6, N-5 and N-Q of the NG samples obtained from the FG samples with different fluorination degrees.

Samples	F/C ratio of FG (at%)	N/C (at%)	N-6/C (at%)	N-5/C (at%)	N-Q/C (at%)
NG’	0	4.91	2.39	1.45	1.07
NG-1	14.32	7.87	5.03	2.49	0.36
NG-2	23.36	11.16	6.60	3.95	0.61
NG-3	31.08	11.99	6.59	3.91	1.49
NG-4	41.97	17.89	10.70	5.69	1.50
NG-5	67.31	28.48	17.01	10.28	1.18
NG-6	93.64	29.09	18.97	8.52	1.60
NG	103.52	29.82	18.49	9.54	1.79

Supplementary Table 5 | The doping levels of total N, N-6, N-5 and N-Q of the N-GQDs samples obtained from the F-GQDs samples with different fluorination degrees.

Samples	F/C ratio of F-GQDs (at%)	N/C (at%)	N-6/C (at%)	N-5/C (at%)	N-Q/C (at%)
N-GQDs’	0	10.18	5.90	5.27	ND
N-GQDs-1	22.17	15.20	8.58	6.61	ND
N-GQDs-2	42.20	18.48	12.00	6.48	ND
N-GQDs-3	77.94	28.64	18.61	10.03	ND
N-GQDs	102.04	36.38	19.92	16.46	ND

Supplementary Table 6 | The doping levels of total N, N-6, N-5 and N-Q of the N-SWCNTs samples obtained from the F-SWCNTs samples with different fluorination degrees.

Samples	F/C ratio of F-SWCNTs (at%)	N/C (at%)	N-6/C (at%)	N-5/C (at%)	N-Q/C (at%)
N-SWCNTs'	0	0.67	0.35	0.32	ND
N-SWCNTs-1	9.94	3.02	1.6	1.02	0.4
N-SWCNTs-2	16.06	3.81	2.19	1.03	0.6
N-SWCNTs-3	18.17	6.32	3.06	2.31	0.94
N-SWCNTs-4	23.59	4.25	2.05	1.41	0.79
N-SWCNTs	34.24	7.79	3.96	3.11	0.72

Supplementary Table 7 | The magnetic properties of graphene and the NG samples obtained at different ammonia annealing temperatures.

Samples	Magnetic coupling	T_c (K)	M_{para} (emu g^{-1})	J	M_{ferro} (emu g^{-1})	M_s (emu g^{-1})
graphene	PM	-	0.14	0.5	-	0.14
NG-400	PM + FM	76.4	1.58	1	0.23	1.81
NG-500	PM + FM	138.2, 250.1	1.99	1	0.31	2.30
NG-600	PM + FM	80.2, 147.0	1.82	1.5	0.18	2.00
NG-700	PM + FM	126.2, 225.2	1.72	1.5	0.17	1.89
NG-800	PM	-	2.98	2	-	2.98

Supplementary Table 8 | The doping levels of total N, N-6 and N-5 of the NG-800 samples obtained by annealing of FG with different fluorination degrees at 800 ^{o}C.

Samples	F/C ratio of FG (at%)	N/C (at%)	N-6/C (at%)	N-5/C (at%)	N-Q/C (at%)
NG-800’	0	3.72	2.07	0.84	0.81
NG-800-1	14.32	10.08	6.12	2.01	1.95
NG-800-2	31.08	13.26	8.50	2.48	2.28
NG-800-3	67.31	15.69	9.32	3.26	3.11
NG-800	103.52	19.24	12.7	3.46	3.08

Supplementary Table 9 | The mass of the pristine, fluorinated and N-doped graphene in the synthesis.

Samples	Annealing temperature (^oC)	graphene (mg)	FG (mg)	NG (mg)
NG-400	400	39.2	72.1	26.4
NG-500	500	33.4	62.6	19.9
NG-600	600	41.3	87.9	23.2
NG-700	700	50.8	101.3	23.5
NG-800	800	52.4	90.0	10.8

Supplementary Table 10 | The mass of the pristine, fluorinated and N-doped graphene in the synthesis. The annealing temperature is 500 ^{o}C.

Samples	Weight ratio of graphene to XeF_2	graphene (mg)	FG (mg)	NG (mg)
NG'	NA	20.1	NA	15.3
NG-1	1:2	49.2	59.4	38.7
NG-2	1:3	45.4	62.2	32.4
NG-3	1:4	46.5	71.8	36.5
NG-4	1:5	48.5	69.4	35.7
NG-5	1:10	44.3	79.5	34.6
NG-6	1:15	47.1	104.4	36.6
NG	1:20	50.1	110.3	35.1
NG"	1:20	43.2	94.7	29.7

Supplementary Table 11 | The mass of the pristine, fluorinated and N-doped graphene in the synthesis. The annealing temperature is 800 ^{o}C.

Samples	Weight ratio of graphene to XeF_2	graphene (mg)	FG (mg)	NG (mg)
NG-800'	NA	31.7	NA	12.2
NG-800-1	1:2	48.5	62.8	15.8
NG-800-2	1:4	49.3	71.1	14.5
NG-800-3	1:10	48.1	85.1	13.6
NG-800	1:20	48.9	108.7	12.9

Supplementary Table 12 | The mass of the pristine, fluorinated and N-doped SWCNTs in the synthesis.

Samples	Weight ratio of SWCNTs to XeF_2	SWCNTs (mg)	F-SWCNTs (mg)	N-SWCNTs (mg)
N-SWCNTs'	NA	48.7		48.1
N-SWCNTs-1	1:5	49.2	48.6	47.5
N-SWCNTs-2	1:10	49.7	56.4	48.3
N-SWCNTs-3	1:20	49.1	59.1	48.6
**Samples**	**Weight ratio of F-SWCNTs-3 to XeF_2**	**F-SWCNTs-3 (mg)**	**F-SWCNTs (mg)**	**N-SWCNTs (mg)**
N-SWCNTs-4	1:20	58.5	58.4	46.4
N-SWCNTs	1:20	59.1	62.7	48.1

Supplementary Table 13 | The mass of the pristine, fluorinated and S-doped graphene in the synthesis.

Samples	Weight ratio of graphene to XeF_2	graphene (mg)	FG (mg)	SG (mg)
SG'	NA	23.4	NA	23.9
SG-1	1:2	24.2	29.4	23.2
SG-2	1:3	25.4	30.9	22.1
SG-3	1:5	25.9	33.1	24.4
SG-4	1:10	24.6	40.6	24.2
SG	1:20	32.4	68.1	30.9

Supplementary Table 14 | The mass of the pristine, fluorinated and S-doped SWCNTs in the synthesis.

Samples	Weight ratio of SWCNTs to XeF_2	SWCNTs (mg)	F-SWCNTs (mg)	S-SWCNTs' (mg)
S-SWCNTs'	NA	34.1		35.7
Samples	Weight ratio of F-SWCNTs-3 to XeF_2	F-SWCNTs-3 (mg)	F-SWCNTs (mg)	S-SWCNTs (mg)
S-SWCNTs	1:20	44.9	48.1	39.1

Supplementary Table 15 | The mass of the pristine, fluorinated and B-doped graphene in the synthesis.

Samples	Weight ratio of graphene to XeF_2	graphene	FG	BG (mg)
BG'	NA	36.9	NA	12.5
BG	1:20	34.3	75.5	14.1

Supplementary Table 16 | The contents of the metal impurity elements (such as Fe, Co, Ni or Mn) of graphene and the NG samples measured by the ICP spectrometry. The unit is ppm.

Samples (ppm)	Fe	Co	Ni	Mn
graphene	10.4	ND	ND	9.2
NG-400	1.5	ND	ND	5.6
NG-500	5.1	ND	ND	5.2
NG-600	34.2	ND	ND	6.6
NG-700	ND	ND	ND	9.4
NG-800	ND	ND	ND	2.1

Supplementary Note 1

As shown in Supplementary Fig. 11a, it is found that no ferromagnetism can be observed in graphene and NG, and only purely diamagnetism can be observed at 300 K. Shown in Supplementary Fig. 11b is the dependence of susceptibility $\chi = M / H$ of graphene on temperature (T) and it fits well with the Curie law $\chi = C / T$. Inset is the corresponding $1/\chi - T$ curve, which demonstrates a linear, purely Curie-like paramagnetic behavior. To analyze the magnetic property of graphene and NG, the $M - H$ curve is fitted by the Brillouin function. As shown by the fitting curve (Supplementary Fig. 11c), the Brillouin function provides good fits for $J = S = 1/2$ for graphene, and the saturated paramagnetization is 0.14 emu g^{-1}. After high-level N-doping, the saturated paramagnetic signal is fitted to be ca. 1.99 emu g^{-1} for NG, and the saturated magnetization of ferromagnetic signal is ca. 0.31 emu g^{-1} (Supplementary Fig. 11d). From saturated paramagnetic magnetization added with saturated ferromagnetic magnetization, one can calculate the M_s of NG is ca. 2.30 emu g^{-1}. To confirm the ferromagnetization of NG, three $M - H$ curves measured at 100, 200 and 300 K were performed (Supplementary Fig. 11e). It is found that NG shows clear ferromagnetism with an obvious coercive field and remnant magnetization at 100 and 200 K, implying that the T_c is above 200 K. Moreover, two clear divergences between FC and ZFC $M - T$ curves emerge at ca. 250.1 and 138.2 K (Supplementary Fig. 11f), revealing further the magnetic transition at T_C.

Supplementary Note 2

Interestingly, it is found that T_c of the NG samples varies (Supplementary Table 7). It may attribute to the difference in the level and type of the N-doping. As reported previously, the N-doping can effectively introduce the magnetic moments[7,8]. As reported in diluted magnetic semiconductors, the ferromagnetic coupling between the magnetic moments may appear via non-localized interaction[9]. Theoretically, the coupling is proportional both to the ordinary Ruderman–Kittel–Kasuya–Yosida (RKKY) oscillation term F which increases with the increase of carrier concentrations, and to the exp(-r), where r is the distance between the magnetic moments. Additionally, N-doping can make the Fermi level shift upward due to the extra π electrons and, thus increase the carrier concentration[10]. In our N-superdoped NG samples, it is reasonable to assume that with the increase of the N-doping level, (i) F will increase with the increasing of carrier concentrations; and (ii) the distance r between the magnetic moments will decrease with the increase of the localized magnetic moments induced by N-doping. As a consequence, the ferromagnetic coupling may enhance and this may result in the increase in T_C.

Furthermore, the bound magnetic polarons (BMPs) may also generate ferromagnetism in semiconductors. Therein, the ferromagnetic coupling can be mediated by BMPs formed by the shallow donor electrons, which overlap to create a spin-split impurity band[11]. Typically, ferromagnetism has been observed in C, Al, or Ca doped ZnO. It is considered that oxygen vacancies can act as BMPs and trap free electrons, and the electrons trapped in these BMPs tend to get easily polarized under the influence of the magnetic field, resulting in ferromagnetism[12]. Note that T_C is strongly related to the BMPs concentration[13]. Similarly, in our case of the N-superdoped NG, the N atoms may act as BMPs thanks to the strong electron affinity[7,14] Thus,

with the increase of the N-doping level, the ferromagnetic coupling may enhance and T_C may increase. Unfortunately, the exact ferromagnetic coupling mechanism is not clear at present, and which need to be confirmed by more experimental and theoretical work.

Supplementary References

1. Araujo, P. T., *et al.* Third and fourth optical transitions in semiconducting carbon nanotubes. *Phys. Rev. Lett.* **98**, 067401 (2007).
2. Nair, R. R., *et al.* Fluorographene: A two-dimensional counterpart of Teflon. *Small* **6**, 2877-2884 (2010).
3. Zhang, C., *et al.* Synthesis of nitrogen-doped graphene using embedded carbon and nitrogen sources. *Adv. Mater.* **23**, 1020-1024 (2011).
4. Robinson, J. T., *et al.* Properties of fluorinated graphene films. *Nano Lett.* **10**, 3001-3005 (2010).
5. Guo, B. D., *et al.* Controllable n-doping of graphene. *Nano Lett.* **10**, 4975-4980 (2010).
6. Liang, J., Jiao, Y., Jaroniec, M. & Qiao, S. Z. Sulfur and nitrogen dual-doped mesoporous graphene electrocatalyst for oxygen reduction with synergistically enhanced performance. *Angew. Chem. Int. Ed.* **51**, 11496-11500 (2012).
7. Liu, Y., *et al.* Realization of ferromagnetic graphene oxide with high magnetization by doping graphene oxide with nitrogen. *Sci. Rep.* **3**, 2566 (2013).
8. Liu, Y., *et al.* Increased magnetization of reduced graphene oxide by nitrogen-doping. *Carbon* **60**, 549-551 (2013).
9. Matsukura, F., Ohno, H., Shen, A. & Sugawara, Y. Transport properties and origin of ferromagnetism in (Ga,Mn)As. *Phys. Rev. B* **57**, R2037-R2040 (1998).
10. Wang, H. B., Maiyalagan, T. & Wang, X. Review on recent progress in nitrogen-doped graphene: Synthesis, characterization, and its potential applications. *ACS Catal.* **2**, 781-794 (2012).
11. Coey, J. M. D., Venkatesan, M. & Fitzgerald, C. B. Donor impurity band exchange in dilute ferromagnetic oxides. *Nat Mater* **4**, 173-179 (2005).
12. Bhosle, V. & Narayan, J. Observation of room temperature ferromagnetism in Ga : Zno: A transition metal free transparent ferromagnetic conductor. *Appl. Phys. Lett.* **93**, 021912 (2008).
13. Yang, S. R. E. & MacDonald, A. H. Disorder and ferromagnetism in diluted magnetic semiconductors. *Phys. Rev. B* **67**, 155202 (2003).
14. Gong, K. P., Du, F., Xia, Z. H., Durstock, M. & Dai, L. M. Nitrogen-doped carbon nanotube arrays with high electrocatalytic activity for oxygen reduction. *Science* **323**, 760-764 (2009).
